# Silicon – single molecule – silicon circuits†

**DOI:** 10.1039/d1sc04943g

**Published:** 2021-10-29

**Authors:** Jeffrey R. Reimers, Junhao Yang, Nadim Darwish, Daniel S. Kosov

**Affiliations:** International Centre for Quantum and Molecular Structures and School of Physics, Shanghai University Shanghai 200444 China Jeffrey.Reimers@uts.edu.au; School of Mathematical and Physical Sciences, University of Technology Sydney NSW 2007 Australia; School of Molecular and Life Sciences, Curtin Institute of Functional Molecules and Interfaces, Curtin University Bentley WA 6102 Australia nadim.darwish@curtin.edu.au; College of Science and Engineering, James Cook University Townsville QLD 4811 Australia daniel.kosov@jcu.edu.au

## Abstract

In 2020, silicon – molecule – silicon junctions were fabricated and shown to be on average one third as conductive as traditional junctions made using gold electrodes, but in some instances to be even more conductive, and significantly 3 times more extendable and 5 times more mechanically stable. Herein, calculations are performed of single-molecule junction structure and conductivity pertaining to blinking and scanning-tunnelling-microscopy (STM) break junction (STMBJ) experiments performed using chemisorbed 1,6-hexanedithiol linkers. Some strikingly different characteristics are found compared to analogous junctions formed using the metals which, to date, have dominated the field of molecular electronics. In the STMBJ experiment, following retraction of the STM tip after collision with the substrate, unterminated silicon surface dangling bonds are predicted to remain after reaction of the fresh tips with the dithiol solute. These dangling bonds occupy the silicon band gap and are predicted to facilitate extraordinary single-molecule conductivity. Enhanced junction extendibility is attributed to junction flexibility and the translation of adsorbed molecules between silicon dangling bonds. The calculations investigate a range of junction atomic-structural models using density-functional-theory (DFT) calculations of structure, often explored at 300 K using molecular dynamics (MD) simulations. These are aided by DFT calculations of barriers for passivation reactions of the dangling bonds. Thermally averaged conductivities are then evaluated using non-equilibrium Green's function (NEGF) methods. Countless applications through electronics, nanotechnology, photonics, and sensing are envisaged for this technology.

## Abstract 2

Single-molecule circuits using silicon contacts are robust, conductive, controllable, and highly reproducible in blinking experiments, with enhanced conductance in break-junctions owing to residual dangling bonds.

## Graphical abstract

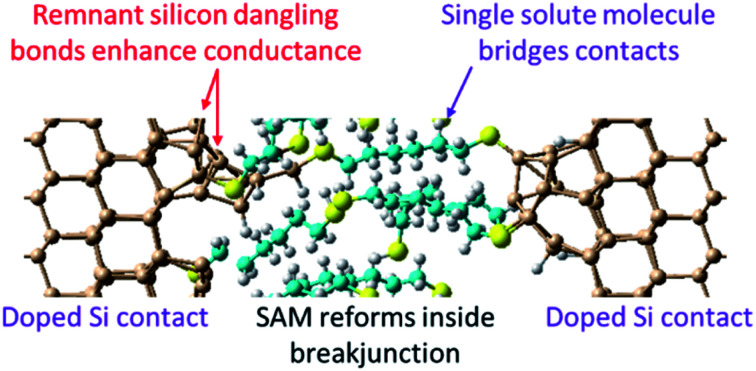

## Introduction

Recently, synthetic techniques compatible with technologies used in silicon fabrication plants were developed that can assemble molecules with either thiol^1^ or disulfide^2^ terminating groups between two silicon-electrode contacts. This can, in principle, pave the way for the inclusion of single-molecules, or else nanoscopic self-assembled monolayers (SAMs), to be imbedded into silicon diodes and transistors. Recently reported conductances for silicon – S(CH_2_)_6_S – silicon junctions were observed to be, on average, one third of analogous values measured on traditional gold – S(CH_2_)_6_S – gold junctions, a useful result. More significantly, the silicon devices displaced mechanical stability lasting 5 times longer, with junction extendibility up to 3 times greater,^1^ and some single-molecule junctions were found to conduct even better than gold junctions. Unlike with traditional gold electrodes, the alignment of the Fermi energy with the molecular orbitals, and consequently the junction's electric properties, can be dopant controlled. Hence these junctions appear attractive compared to standard approaches used in Molecular Electronics.

A key feature of the synthesis conditions recently developed^1,2^ is that they require no heating, pressure, irradiation, or external catalysis. This differs from the wide variety of approaches previously used for grafting molecules onto silicon^3^ using, *e.g.*,^4–6^ Lewis acids,^7^ Grignard reagents,^8,9^ electrografting,^10^ and microwave^11^ or UV-Visible irradiation,^12–18^ involving perhaps ultra-high vacuum technologies,^19,20^ high-temperature solution chemistry,^21,22^ or high-temperature high-pressure processes in supercritical CO_2_.^23^ Techniques that form oxide-free surfaces have been of central importance,^8,9,23–30^ and the newly developed techniques not only avoid oxide but also avoid fabrication-induced SAM damage.

Utilising control over both the properties of the silicon contacts and the bridging molecule,^8,28,30–33^ applications can be envisaged to field-effect transistors^25,30,34,35^ perhaps for biomedical applications,^29^ electrochemical applications^36,37^ including sensing,^38^ polymer engineering,^39^ hydrophobicity,^40^ quantum-dot photonics,^41^ photoluminescence,^42^ light harvesting and usage,^43,44^ bioimaging, biosensing, and cancer treatment,^45,46^ as well as molecular-electronics applications.^31,36,47–51^ Silicon–molecule–metal junctions can also be envisaged and would have useful properties, by analogy to results found for GaAs–molecule–Au junctions.^52–54^

Thiol SAMs on gold have dominated previous applications in molecular electronics as they are also easy to prepare and have properties of widespread interest,^32,55–62^ but suffer from drawbacks as the Au–S bonding is weak^63^ and dispersion controlled.^64–66^ To make robust devices of atomic dimensions, structural regularity and stability is important, and hence covalent bonding of molecules to silicon offers new technology directions; related covalent-bonding applications involving, *e.g.*, graphene point contacts are also of modern interest.^67–69^

**Fig. 1:**
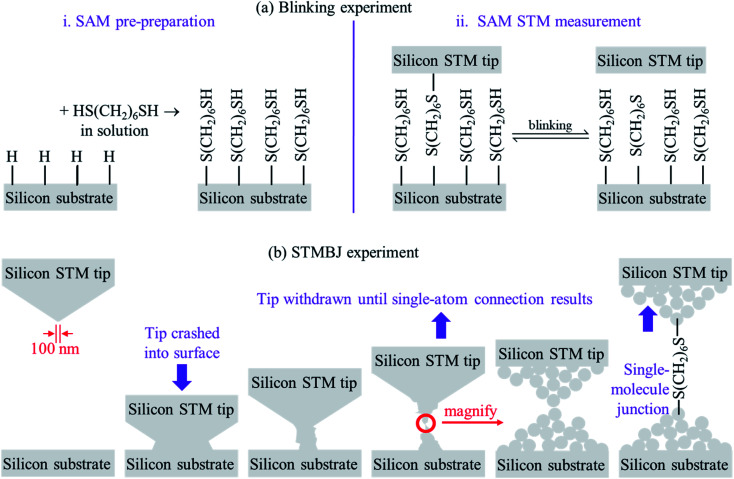
Sketches of (a) the blinking experiment, and (b) the STMBJ experiment. Blinking experiments are performed on pre-formed regular SAMs (at *ca*. 75% coverage), bringing up an STM tip that is assumed to be flat on the atomic scale, detecting single-molecule junctions forming and breaking. STMBJ experiments involving crashing an STM tip into the substrate, and withdrawing it until first single-atom-wide silicon–silicon junctions form and then further until single molecules from solution bridge the formed gap.

To date, two types of silicon – molecule – silicon junctions have been prepared using scanning-tunnelling microscopy (STM) technology: “blinking” junctions, formed by holding a silicon STM tip fixed above a SAM of the molecule pre-prepared on a Si(111)–H substrate, and “break-junction” (STMBJ) junctions formed by crashing a silicon tip into a silicon substrate and then withdrawing the tip. Fig. 1 illustrates the two approaches. The blinking method is adapted from the current–time approach of Nichols and co-workers,^70^ whilst the STMBJ method is adapted from the approach of Tao and co-workers.^71^ Such approaches drive many modern applications in Molecular Electronics.^72^

The blinking junctions (Fig. 1a) are so-called as the connection between the bridging molecules and one of the electrodes can break and reform on the seconds timescale. The STM tips used in such experiments are regarded as being “sharp”, yet present curvatures of the order of 100 nm and hence can be considered as being atomically flat on the length scale of the experiment. Note that mostly one molecule at a time bridges the two silicon electrodes, despite the large surface area of interaction. The blinking experiment is therefore one in which the atomic structures are controlled: the SAM is ordered, and the two silicon surfaces are ordered. Experimental conditions can be varied to control factors such as SAM coverage and structure.

On the other hand, understanding the atomic structure of STMBJs involving silicon (Fig. 1b) demands solution of a significant number of issues. When the tip is crashed into the substrate, damage to both structures over multiple atomic layers is expected. The original Si(111)–H surface termination will be destroyed by the impact. Covalent bonds will form between the atoms originally in the tip and those originally in the substrate, and extraction of the tip will result in the fracturing of enough covalent bonds to allow separate structures to reform. This will expose silicon atoms on both tip and substrate with “dangling bonds” associated with the collision and separation processes. As these experiments are performed in solution, dangling bonds will then react with re-entering solvent and/or solute, plus any contaminant molecules that it may bring. Relevant to this work, STMBJ conditions involve 1,3,5-trichlorobenzene as the solvent, with the 1,6-hexanedithiol reactant present at 4 μM concentration.^1^ The timescale of individual STMBJ measurements is of the order of ms, and so to modify outcome, such reactions must be completed by then. Reported experimentally is conductance information averaged over thousands of such collisions.

This work concerns, firstly, the elucidation of the atomic structure of Si – S(CH_2_)_6_S – Si junctions formed under either blinking or STMBJ conditions. Some basic interfacial structural models are postulated and optimised using density-functional theory (DFT), mostly utilising molecular dynamics (MD) simulations at 300 K.^73^ Structures considered include: simple SAMs on regular silicon-surface structures with or without hydrogen termination; fractured silicon structures after STMBJ tip retraction; transition-state structures for reaction of dangling bonds with solvent, solute, and possible contaminants; and SAMs reformed to fractured silicon structures after tip retraction. These structures are obtained as a function of gradual retraction of the STM tip. They are then tested using DFT-based non-equilibrium Green's function (NEGF) calculations^74^ of the electric properties of the electronic device, partitioned as semi-infinite doped silicon – junction region – semi-infinite doped silicon, leading to the evaluation of the electrical conductance. How this conductance varies as a function of tip retraction is then compared with observed conductances,^1^ allowing the principles that lead to the observed exceptional device characteristics to be determined. Such knowledge of atomic structure and dynamics, electronic structure, and conductivity is essential for the design and interpretation of almost all experiments performed on applications systems and devices. NEGF calculations are performed for the situations in which the silicon tip and substrate are P-doped (1.15 × 10^20^ atoms cm^−3^) and N-doped (7 × 10^19^ atoms cm^−3^) – these doping levels correspond to the same silicon bulk resistivity of 0.001 Ω cm.^75^

## Results

### (a) Structure and conductivity of the regular SAMs examined in blinking experiments

**Fig. 2:**
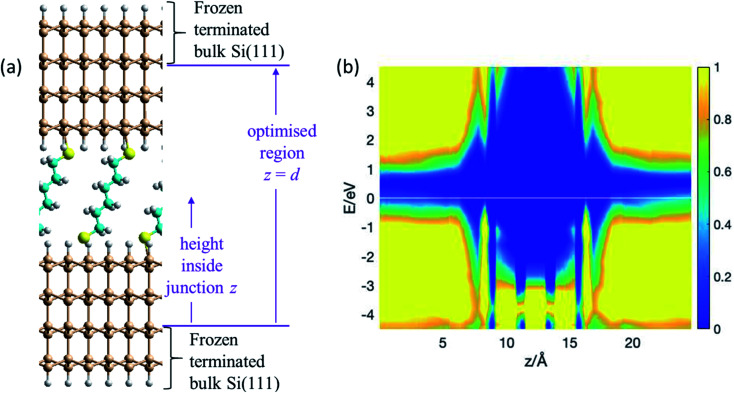
Modelling the blinking experiment. (a) 2D (3 × 3) model of two flat Si(111)–H surfaces spanned by S(CH_2_)_6_S at 1 : 9 coverage; bulk silicon is emulated by H-terminating the outer layer of each silicon slab, with this and the next inner layer frozen at bulk coordinates. The vertical height inside the junction is taken to be the distance *z* from the uppermost frozen atom in the lower electrode. The junction length is taken to be the vertical extent, *d* = 24.6 Å, of the region containing optimised atoms. Si-brown, S-yellow, C-cyan, H-white. (b) PDOS (in (eV Å)^−1^) from NEGF calculations of the conductivity, showing the relative density of electronic states (colour coded) as a function of the vertical height *z* inside the junction and the electron energy *E*. See also ESI† Fig. S1.

The atomic model used for the blinking experiments involves a single S(CH_2_)_6_S group bridging regular Si(111)–H surfaces representing the substrate (lower surface) and tip (upper surface). Owing to the large curvature of the STM tip (Fig. 1), the upper electrode is assumed flat on the atomic length scale, and the same surface model is therefore used for both surfaces. The thiol hydrogen atoms are regarded as being lost by the chemisorption process^1^ that binds the solvent molecule to the two surfaces. Structures are obtained by taking an already-prepared^1^ low-coverage (1 : 9) SAM of S(CH_2_)_6_SH on (3 × 3) Si(111)–H, removing the tail thiol H atom, and optimising the coordinates obtained when a second Si(111)–H surface is brought up that contains one silicon dangling bond. The optimised structure is shown in Fig. 2a; full structural and computational details for this, and indeed all structures reported in figures, are provided in the ESI.†

NEGF calculations of the conductance of this blinking-experiment model yields transmission curves (see Fig. S1 (ESI†)) and projected densities of states (PDOS, Fig. 2b) for the situations in which the silicon tip and substrate are P-doped and N-doped. The PDOS tells the electronic state density at height *z* above the start of the geometrically optimised region of the junction and at electronic energy *E* away from the Fermi energy. Fig. 2b shows the PDOS for P-doped silicon, for which the Fermi energy is at the top of the valence band. Within the molecular region, features in the PDOS are seen at *z* = 8.3 Å and 16.3 Å arising from the sulfur atoms. Between them are three bands each attributable to two carbons. This electronic structure can interact with the valence band of the silicon and portrays a significant conduction pathway that is close to the Fermi energy, supporting hole conductivity in the P-doped junction. As Fig. 2b implies and ESI† Fig. S1 details, no such analogous pathway for electron conduction is apparent, implying that the junction conductance will be much greater for P-type Si than for N-type. Indeed, our NEGF calculations predict a ratio of 10 : 1 for the conductance in P-type silicon compared to N-type.

Fig. 2b depicts the electronic properties of the electrodes and bridging molecule that are critical to the ability to perform realistic conductance predictions. However, as one sees from the figure (and also ESI† Fig. S1), the native silicon bandgap is predicted to be 0.6 eV, significantly removed from the actual value^76^ of 1.17 eV. The calculations could by enhanced by using either improved density functionals,^77^ or GW-based NEGF theory^78,79^ to open up the silicon bandgap; however, these calculations are hardly feasible for the size and number of geometries considered herein. Subtle but sometimes significant effects^80^ associated with the stabilisation of (partial) molecular charges at the interface are also not included in these calculations. The difficulty in making quantitatively reliable predictions of junction conductance are likely to be enhanced when irregular and unterminated silicon contacts are considered in subsequent results subsections.

To date, conductance for this junction has only been measured^1^ for P-type Si, with a summary given in Table 1. Those experiments were performed using boron P-doped silicon with a bulk resistivity of 0.001 Ω cm, which corresponds to the dopant concentration used in the modelling. The observed conductance at zero voltage is on average *G* = 70 μ*G*_0_, where *G*_0_ = 2*e*^2^/ℏ = 77.5 μS is the quantum of conductance. At half maximum, the conductance range HM_−_–HM_+_ is 65–85 μ*G*_0_, a narrow range with ratio *η* = HM_+_/HM_−_ = 1.3. In comparison, the calculated conductance is 4 μ*G*_0_; an order of magnitude difference between observed and calculated conductances is indicative of reasonable qualitative agreement.

**Table 1:** Comparison of observed^a^ and calculated conductances (μ*G*_0_) at zero voltage for P-type silicon and for gold junctions bridged by S(CH_2_)_6_S

Junction	Calc.	Peak	HM_−_	HM_+_	*η*
Si blinking	4	70	65	85	1.3
Si STMBJ	28^b^	60	18	250^c^	14.3
Au blinking	230 (ref. 1)	180	150	210	1.5
Au STMBJ	230^d^	200	90	350	3.9

a Peak, values at half the maximum on the high (HM_+_) and low (HM_−_) sides, and their ratio *η* = HM_+_/HM_−_, extracted from the probability distribution^1^ of log(*G*/*G*_0_) so that the peak corresponds to the geometric mean of the observed conductances.

b Geometric mean from Fig. 6e at 300 K.

c Long tail, perhaps extending beyond the detection limit of 1000 μ*G*_0_.

d At *T* = 0 K.

A gauge of the usefulness of the calculated result is its comparison with analogous data pertaining to HS(CH_2_)_6_SH tethered between gold electrodes. The calculated conductance at zero voltage is 230 μ*G*_0_, whilst the observed^1^ conductance is similar, displaying a peak at 180 μ*G*_0_ and a range of 150–210 μ*G*_0_ (*η* = 1.5). Of note, the narrower observed conductance range for silicon junctions (*η* = 1.3) implies that the blinking silicon junctions are more reproducible and more stable than the analogous gold junctions. Concerning the calculations, increased errors are anticipated for the modelling of silicon junctions compared to metal junctions as results will depend significantly on how doping, band-gap, and other effects are handled within the DFT and NEGF calculations.

### (b) A crude atomic model of an STMBJ: small H-terminated tips on regular Si(111)–H surfaces

Little is known about the atomic structures prepared after a silicon STM tip is crashed into a silicon substrate and then withdrawn, as occurs in an STMBJ experiment. Nevertheless, the unique properties of silicon STM tips after they are crashed into a surface and retracted have long been exploited to make high-resolution STM images,^81^ with retracted tips shown to retain function on the day's timescale. It is likely that the differences between standard silicon STM tips and such “super tips” are captured in the caricatures of the blinking and STMBJ experiments presented in Fig. 1. For silicon tips produced by surface collisions, allowed to equilibrate and then used in subsequent experiments, the assumption of small apex-shaped H-terminated tips has led to useful modelling of high-resolution STM images,^82^ and hence this model is taken as a starting point.

**Fig. 3:**
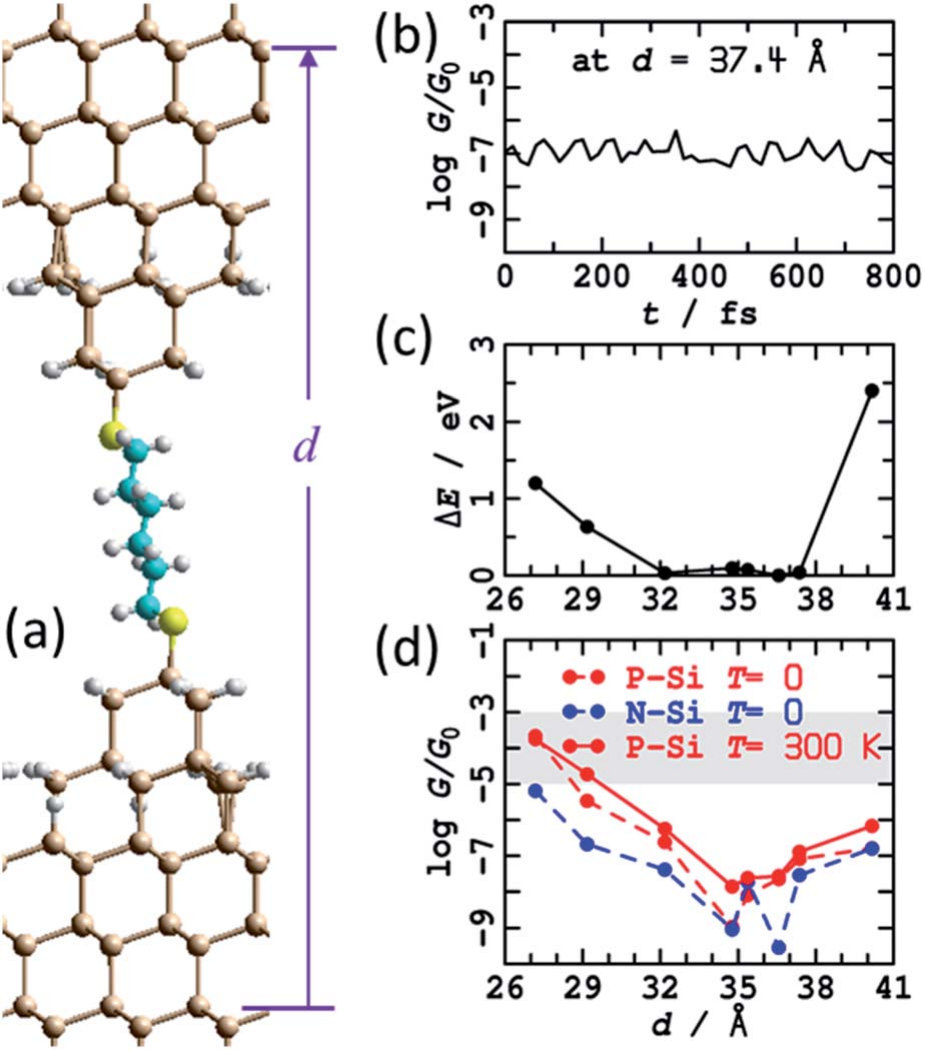
Modelling the STMBJ experiment assuming regular H-terminated silicon tips form on each electrode. (a) 2D (3 × 3) model of two flat Si(111)–H surfaces with two added H-terminated Si layers in the shape of a tip, spanned by S(CH_2_)_6_S, at *d* = 36.6 Å; Si-brown, S-yellow, C-cyan, H-white. (b) Conductivity at zero voltage evaluated along a 800 fs MD trajectory for P-type silicon. (c) Average energy of each MD simulation. (d) Conductivity at zero voltage for P-type and N-type silicon for static geometries (*T* = 0 K) and averaged over *T* = 300 K MD trajectories; the shaded region shows the observed conductance found throughout extensions of 3–10 Å for P-type Si at 300 K. See ESI† Fig. S2 and S3 for more information.

In Fig. 3 is shown the atomic model used to depict this scenario. It consists of regular two-layer tips (9 and 4 Si atoms in each layer) that are fully H-terminated and sit on top of Si(111)–H surfaces. The horizontal alignment of the top and bottom electrodes is adjusted to minimise the energy. This is done by first optimising the coordinates of a constrained atomic-cluster model,^83^ then mapping out a potential-energy surface as a function of the separation between the tips, see ESI† Fig. S2. Overall, this separation is varied over 13 Å, manifesting junction structures that present at least five distinct conformations of the alkane chain. Eight representative structures from this cluster model were then inserted into 2D periodic models of the junction and re-optimised, freezing the outside two layers of each silicon electrode at 3^rd^-layer (from the surface) to 3^rd^-layer distance *d*, see Fig. 3 and ESI† Fig. S3.

A primary result apparent in Fig. 3 is that, through alkane conformational changes and bond-extension possibilities, these STMBJs can form over 13 Å length extension. This is consistent with key experimental results^1^ depicting junctions stable over typically 3–10 Å. Contrary to experiment, however, the calculated conductances are shown in Fig. 3c and depict four orders of magnitude variation in the conductance. Observed^1^ conductance histograms (of log(*G*/*G*_0_)) depict circuits formed with *G* = 10–1000 μ*G*_0_, with a conductance maximum (approximate geometric mean) at 60 μ*G*_0_ and conductances at half-maximum of 18 and 250 μ*G*_0_ (Table 1) giving *η* = 14. To compare with the logarithmic scale reported in Fig. 3 and subsequent figures, the observed full conductance range is −5.0 < log_10_(*G*/*G*_0_) < −3.0, and representative shaded regions are drawn on the figures.

In summary, the calculated conductances for the regular H-terminated tip model are too low and too variable to explain the observed conductance plateaus. It also appears unlikely that STMBJ structures of this type on regular structures could account for the very wide range of conductances observed in the experiment (*η* = 14, Table 1) and its sharp contrast to that observed in blinking experiments (*η* = 1.3). In traditional STMBJ experiments using gold electrodes, it is customary to expect greater conductance variations in STMBJ experiments, *e.g.* for HS(CH_2_)_6_SH, *η* = 1.5 in STMBJ experiments compared to 1.3 in blinking ones (Table 1),^1^ but the effect using silicon is dramatically magnified, increasing from 4 to 14.

Nevertheless, insight gained by consideration of the calculated conductances for the regular H-terminated junction provides important information needed to understand more complicated junction models. ESI† Fig. S3b shows transmission as a function of extension for thus model. The conductivity variation shown in Fig. 3d throughout 27 Å ≤ *d* ≤ 35 Å arises from direct through-space tunnelling between the silicon electrodes (see also ESI† Fig. S5); it takes a similar form if the bridging molecule is removed and the conductance calculations simply repeated. Within the range 35 Å ≤ *d* ≤ 40 Å, the conductance increases owing to alkane-chain conformational straightening. The conductance is predicted to increase significantly as *gauche*-type conformations are eliminated at larger junction extensions.^73^ To interpret the small observed variations in conductance over extensions of 3–10 Å, it is apparent that only straight-chain conformations, or conformations with at most one *gauche* linkage, can be involved. Hence structural variations other than chain conformational changes must be responsible for a substantial part of the observed effect.

The relatively high conductances indicated in Fig. 3d at *d* = 40 Å arise as the molecule has become highly stretched. This reduces the molecular band gap and hence increases the conductivity. In practice, such effects may not be observable as stretched structures have a high energy cost of production (Fig. 3c), and hence are likely to embody unsustainable force magnitudes. Indeed, the average force between the last two data points in Fig. 3c is 0.7 eV Å^−1^, larger than the value of ∼0.5 eV Å^−1^ observed^84^ for physisorbed sulfur–gold junctions. Hence, of the structures considered, the ones that are most indicative of through-molecule conductivity through linear (or near linear) chains are those in Fig. 3d in the range of 35–37.4 Å. These are 100 times smaller than those calculated for the blinking experimental configuration. Added fully H-terminated tips therefore act as insulators that block conductivity from the silicon through the molecule.

In general,^85^ molecules can show considerable enhancements of conductance between metal electrodes using STMBJ experiments compared to blinking experiments, although much small effects are common,^86–89^ and specifically the effect for HS(CH_2_)_6_SH between gold electrodes is quite small (Table 1). Complementary calculations for junctions with Au electrodes were performed, with the conductance calculated for HS(CH_2_)_6_SH between flat Au surfaces being *G* = 227 μ*G*_0_, compared to 229 μ*G*_0_ when 4-atom tips are inserted on each side of the junction (Table 1). The relative insensitivity to interface structure can be attributable to the ability of gold SAMs to adapt to their circumstances,^90–92^ although for some properties such as inelastic electron-tunnelling spectroscopy (IETS) structural details can be critical.^93^ That the calculations predict 100 times reduction in conductance for regular H-terminated silicon STMBJ tips therefore presents strong evidence suggesting that such a chemical model is inappropriate, a conclusion supported by the observed large conductance range (*e.g.*, *η* = 14, Table 1).

One effect of note is that the experiments are performed at room temperature, whereas the junction structural models so far reported pertain to 0 K and do not include thermal fluctuations of the interface. The eight representative structures shown Fig. S3a† (and Fig. 3a) are actually those following 1 ps of MD at 300 K. Shown in Fig. 3b is the conductance calculated for the (perhaps most important) trajectory at *d* = 37.4 Å. The predicted variation in conductance attributable to thermal fluctuations is small compared to other effects that have been noted. As a function of junction extension, the calculated conductances at 0 K and 300 K are very similar, see Fig. 3d. These results are consistent with the identification of the structure of “super tips” used in high-resolution STM imaging as having this basic structural form.^81,82^

### (c) A crude atomic model of an STMBJ: small bare tips on regular Si(111) bare surfaces

**Fig. 4:**
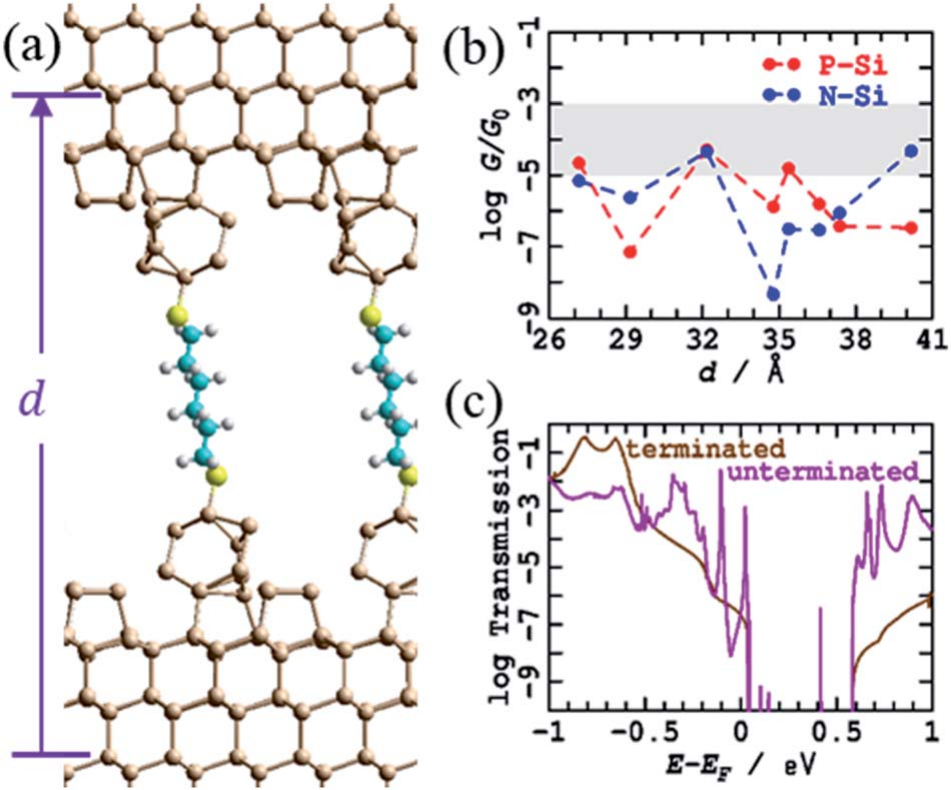
Modelling the STMBJ experiment, using regular unterminated silicon tips on each unterminated electrode. (a) 2D (3 × 3) model for tips spanned by S(CH_2_)_6_S at *d* = 37.37 Å; Si-brown, S-yellow, C-cyan, H-white. (b) Conductivity at zero voltage for P-type and N-type silicon at 0 K; the shaded region shows the observed conductance found throughout extensions of 3–10 Å for P-type Si. See ESI† Fig. S4 for more information. (c) Comparison of the transmission for P-type Si, as a function of electron energy from the Fermi energy, at *d* = 32.17 Å, from this unterminated series to that for the analogous terminated structure (Fig. 3, ESI† Fig. S3).

A variant crude structural model was then used in which the bare silicon tips produced after breaking of the contacts in an STMBJ experiment (Fig. 1) are assumed not to react extensively with solvent or solute on the ms timescale of the experiment, leaving all dangling bonds bare. The simplest model for this is to take the H-terminated surface and tips from Fig. 3, remove the terminating hydrogens, and re-optimise the structures. This was followed by MD simulations for 1 ps at 300 K, an operation that induced significant structural changes to the interface, see Fig. 4a for *d* = 37.4 Å; results at other tip extensions are shown in ESI† Fig. S4. As shown in Fig. 4b, conductances at most junction separations are significantly enhanced compared to the analogous fully H-terminated model in Fig. 3, often now encroaching on the region in which conductance is observed to be sustained over 3–10 Å extensions. Nevertheless, the conductance is predicted to change erratically over 5 orders of magnitude during tip extension, differing from experiment^1^ not only in the range of the variation but also in that individual conductance traces are usually observed with at most factors of 3 variation.

Fig. 4c demonstrates the cause of this erratic behaviour, the major qualitative difference found between calculations using regular tips with and without H-termination. Shown is the electronic transmission probability as a function of energy away from the Fermi energy for P-type silicon at *d* = 32.2 Å, evaluated for both H-terminated tips and surfaces and for their unterminated analogues. For the unterminated tip, silicon dangling-bond states appear within the silicon bandgap at seemingly random intervals. If perchance one of these states appears close to the Fermi energy and the molecular energies, then it will dramatically enhance conductivity, giving rise to the profound differences in conductivity perceived between Fig. 3d and 4b, as well as the erratic tip-extension dependence shown in Fig. 4b.

The variation in the electronic structure associated with dangling bonds will be very sensitive to the details of both the silicon geometry and the silicon–sulfur interface. Hence the conductance is expected to vary significantly during MD simulations. Realistic calculations of junction conductance therefore require understanding of what actual tip shapes could be, how these can react with solvent, solute, *etc.* on the experimental timescale, and understanding of the thermal effects that are likely to modulate them. Needless to say, quantitatively accurate calculations will also require a much better description of the silicon band gap than the current, PBE-based, calculations provide.

Inspiration concerning the basic nature of the problems encountered in understanding the nuclear and electronic structure of unterminated Si(111) can be found in the literature of the bare Si(111) surface itself. For this, following decades of research, controversy remains concerning the basic surface nuclear and electronic structure, as well as the determination of appropriate computational methods for its simulation.^94,95^ The central issue pertains to how silicon dangling bonds interact with their neighbours, concerning both the alleviation of the intrinsic chemical instability that arises and the resulting electronic structure and its influence on conductivity. Unless full passivation of the STMBJ tips occur after the solution re-enters the broken junction, such issues will also arise. As full passivation acts as an electrical insulator (Fig. 3), it is unlikely to have been observed in silicon STMBJ experiments.

### (d) Structures made using MD to fracture a fused substrate-tip structure

In search of a more realistic silicon atomic topology, MD simulations were performed, at 300 K, in which an initial structure representing the STM tip crashed into the substrate was taken and pulled to breaking point. The initial structure, somewhat unrealistically, was taken as an 11-layer Si(111) slab with random atoms removed from the central layer. An odd number of total atoms was chosen to assure asymmetry in the products obtained. Steps during the fracturing process, as well as in its compression to the bulk silicon density, are illustrated in ESI †Fig. S5. These MD simulations do not take into account any large-scale tip deformations that could occur on the experimental timescale^85^ as they explore only the chemical processes occurring at the junction interface. The conductance as a function of the fracturing is also shown in ESI† Fig. S5.

**Fig. 5:**
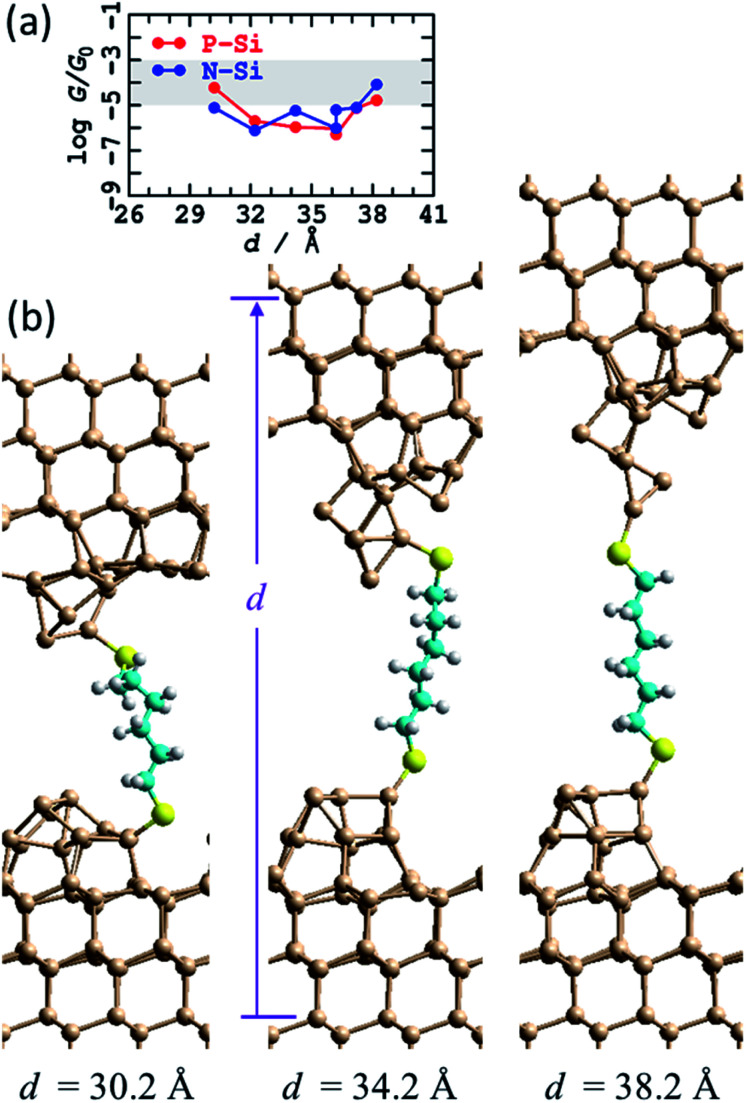
Modelling the STMBJ experiment, following MD at 300 K on regular unterminated silicon tips made by simulating a tip-surface crash and withdrawal (see ESI† Fig. S5). (a) Conductivity at zero voltage for P-type and N-type silicon at 300 K; the shaded region shows the observed conductance found throughout extensions of 3–10 Å for P-type Si. (b) 2D (3 × 3) model for tips spanned by S(CH_2_)_6_S; Si-brown, S-yellow, C-cyan, H-white. See ESI† Fig. S6 for more information.

The two irregular tips so produced, one with a clear apex atom, the other with 3 atoms in its uppermost plane, were not H-terminated and a straight-chained single bridging unit S(CH_2_)_6_S was added to connect to the top and bottom electrodes. MD calculations were then run for 1 ps, and the structures were slowly pulled apart and the dynamics repeated for each step (Fig. 5b). At the shortest separation investigated, a kink resulted in the alkane chain, but otherwise a linear chain persisted over an 8 Å retraction of the top electrode. To facilitate this, a binding location change occurred, with the S atom binding to an Si atom below the top of the tip at short distances, and to the top at larger ones.

Fig. 5a shows that this model predicts conductances that strongly encroach on the observed range, sustained for extensions of up to 8 Å as also observed. These were averaged over 1 ps of MD and do not show the same erratic behaviour found in Fig. 4 as the accidental resonances between dangling-bond levels and molecular levels that drive conduction are averaged over. From a quantitative perspective, the thermal fluctuations in the conductance are so large that the performed MD averaging is insufficient, and hence order-of-magnitude error bars may still be anticipated for the thermally averaged results.

### (e) Likely chemical reactions on the fractured structures

As dangling bonds dominate conduction properties, it would seem essential that the best-possible descriptions of the nuclear and electronic structures of STMBJ junctions be obtained. This demands understanding how dangling bonds become passivated on the ms STMBJ timescale.

In the experiment being modelled, exposed silicon dangling bonds would react rapidly with any dissolved O_2_ in the solution to form surface oxides. The facile synthesis used to make the junctions starting from thiol reactants relies on the presence of ambient levels of dissolved O_2_, which effectively provides a catalyst for the process.^1^ In this way, all dissolved O_2_ is believed to be consumed, and indeed no trace of surface oxide can be found after SAMs were left standing. Hence the available experimental evidence indicates that oxide formation by this mechanism is not a likely process.

An alternative route to surface oxides is through reactions of dangling bonds with contaminate water in the 1,3,5-trichlorobenzene solvent. The solvent had been extensively dried,^1^ but the concentration of the dithiol solute is only 4 μM and so water may still present possible reactive pathways. According to standard bond-energy tables, the reaction

1 2Si˙ + H_2_O → SiOH + SiH

should be exothermic by 3.1 eV. A MD simulation was run in which the gap between the two unterminated electrodes was widened and liquid water inserted, see ESI† Fig. S7. Water reacted with the dangling bonds within 1 ps. Hence the water concentration is an important parameter pertaining to the chemical stabilisation of the bare tips.

The solute molecules HS(CH_2_)_6_SH clearly do react with the silicon tips on the STMBJ timescale as the observed conductance signal stems from the resultant bridged-electrode structure. Calculations^1^ predict that thiols react barrierlessly with silicon surface radicals, hence facilitating sub-ps reaction times akin to that calculated above for the reaction with water. At 4 μM concentration, the reaction time would be expected to be in the ns–μs range. Diffusion of solvent and solute into the nano-cavity formed by tip retraction is therefore likely to be a critical aspect in determining the actual reaction kinetics. The relative importance of water reacting instead of the dithiol would scale with the relative concentrations of the water contaminant to the dithiol solute. If the reaction with water was dominant, then break junctions would be difficult to form. Hence the experiment suggest that reactions with water are not significant, and we neglect this possibility henceforth.

A reaction of surface dangling bonds was noted in some MD runs when the starting geometry was at high energy. This involved the hydrogen abstraction reaction

2 2Si˙ + HS(CH_2_)_6_SH → 2SiH + HS(CH=CH)(CH_2_)_4_SH,

but standard bond energies suggest this reaction is endothermic and therefore not of great concern.

Reactions with the 1,3,5-trichlorobenzene solvent provide another possible mechanism to stabilise surface silicon dangling bonds. Some products obtained computationally by bringing a solvent molecule up to either the top or bottom tip fragments from ESI† Fig. S5 are shown in ESI† Fig. S8. A reaction to break a C=C π bond in the benzene and add two Si–C bonds in its place was calculated to be exothermic, with Δ*E* = −0.39 eV, whilst the insertion of a Si biradical inside a C–Cl bond was calculated to be more so, with Δ*E* = −1.70 eV. A subsequent H-shift onto a neighbouring Si radical lowered the energy further to −2.88 eV. Analogous insertions of a silicon biradical into a solvent CH bond were predicted to be endothermic. All reactions considered were found to require activation, with the lowest-energy transition-state obtained being that for the Si biradical insertion reaction at Δ*E*^†^ = 0.49 eV. To be competitive against a barrierless reaction with the solute at 4 μM concentration, the reaction barrier would need to be less than 0.3 eV.

Of all the reaction mechanisms considered, the most likely one under the experimental conditions is therefore reaction of silicon radicals with the dithiol solute molecules. This reaction is expected to be fast on the ms timescale of the experiments, and is known to occur to some extent as only the products of this reaction can give rise to the observed conductivity. Whilst the calculations do not rule out the possibility of reactions of silicon dangling bonds with the solvent, this possibility is neglected in the subsequent modelling.

### (f) Molecular dynamics to model reformed SAMs

**Fig. 6:**
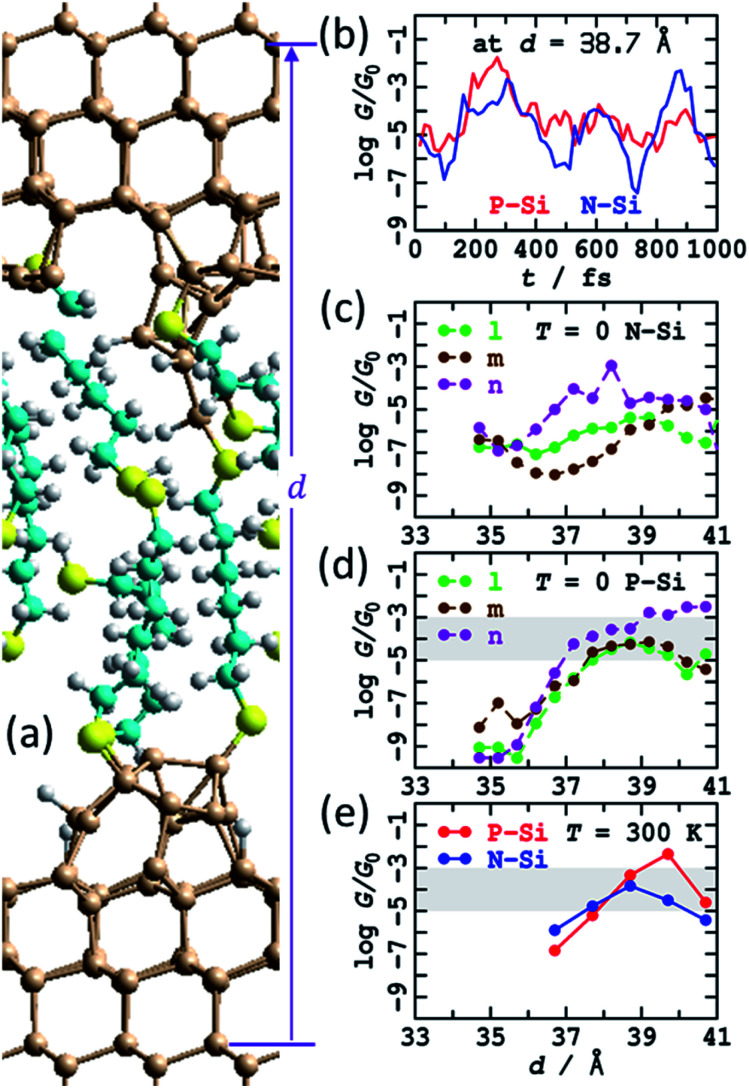
Modelling the STMBJ experiment, following MD at 300 K on SAMs made by simulating a tip-surface crash and withdrawal and then chemisorption of HS(CH_2_)_6_SH solute molecules, with one molecule bridging top and bottom. (a) 2D (3 × 3) model for tips spanned by S(CH_2_)_6_S; Si-brown, S-yellow, C-cyan, H-white. (b) Conductivity variations at zero voltage for P-type and N-type silicon at 300 K along a MD trajectory. (c) Conductivity at zero voltage for P-type silicon at 0 K. (d) Conductivity at zero voltage for N-type silicon at optimised geometries (*T* = 0 K). (e) Conductivity at zero voltage for P-type and N-type silicon averaged over *T* = 300 K MD trajectories. The shaded region shows the observed conductance found throughout extensions of 3–10 Å for P-type Si. See ESI† Fig. S5 and S9–S12 for more information.

The calculations suggest that the bare silicon tips formed after fracturing of a crashed tip and substrate junction will spontaneously react with the dithiol solute molecules to reform SAMs on both the top and bottom electrodes. Three possible SAMs attached to the fractured tips from Fig. S5† were manually constructed and named “l”, “m”, and “n”. This construction was done at a short electrode spacing such that the SAMs attached to the top and bottom electrodes overlapped, delivering a combined SAM coverage of 6 : 9, slightly less than the value of 75% ± 8% observed^1^ for dense regular S(CH_2_)_6_S SAMs on flat Si(111)–H surfaces. Results following optimisation at 0 K are shown in see Fig. 6 and ESI† Fig. S9a–c, including calculated conductances in both N-type and P-type silicon. The structures were then stretched and the geometry optimisations repeated. The total extension applied was 6.5 Å, encompassing some alkane-chain conformational changes at short distances and junction rapture at the largest separation used, *d* = 41.2 Å. The calculated conductances shown in Fig. 6b and c show regions of regular conductance extending over 4 Å of stretching, with magnitudes similar to those observed. Combined, the three series motivate how seemingly similar chemical structures can sustain conductance over the lower-region of the range observed, with conductance values spreading over the wide observed range.

The “n” series was then chosen and MD simulations run for 1 ps. Sample structures obtained are highlighted in ESI† Fig. S10; at large distances, fracturing of the junction, at least for small times, are predicted, sometimes associated with Si–S bond breakage and sometimes with Si–Si cleavage. Sometimes, dynamics resulted in chemical reactions between the breaking fragments and the surrounding SAM, forming S–S or Si–S bonds, indicating a new mechanism for chemical reactions occurring inside molecular junctions.^89,96,97^ Averaged conductances are reported in Fig. 6e as a function of extension and overall in Table 1 (900 μ*G*_0_), many averaged junction properties are reported in ESI† Fig. S12, and individual conductance time histories are reported in ESI† Fig. S11 and Fig. 6b. The variations in conductance with distance averaged over the MD trajectories appears enhanced compared to those reported in Fig. 6c and d for *T* = 0, though the sampling performed may not be sufficient to justify this conclusion. Indeed, the calculated time dependence of the conductivity is large (Fig. 6b, ESI† Fig. S10), as found for previous MD simulations. The number of passivated dangling bonds per cell in these junctions is 14, whereas the number of remnant dangling bonds is 18, so only 43% are passivated, and the unpassivated bonds continue to exert a dominant influence over the conductivity.

Note that none of the investigated structures could facilitate slippage of the Si–S contact from an inner Si row to an outer row, as was found in Fig. 5 to significantly enhance the extendibility junctions beyond the *ca*. 4 Å extent perceived in Fig. 6. Another limitation of the current calculations is that they do not allow for extended surface passivation by SAM growth as the tips are pulled apart and the overlap between the SAMs bound to the top and bottom electrodes diminishes. Such SAM growth would act to reduce the coverage of dangling bonds, but the coverage would presumably remain sufficient to maintain the predicted high conductances.

## Conclusions

The calculations presented herein confirm the observed relatively high average conductivity of the silicon junctions, explain how it can be that some silicon junctions can be more conductive than typical gold ones, and reveal the mechanisms through which the increased extendibility arises: a combination of Si–S bond-slippage effects, as well as Si-tip reconstruction and elongation, rather than alkyl-chain conformational flexibility. Overall, the calculations motivate how it is that STMBJ junctions can display conductances varying over an extremely wide range, and that conductance can be sustained over unprecedented mechanical extensions.

Whereas specific quantitative predictions arising from the calculations performed are likely to be wayward owing to the many simplifications used, the overall qualitative scenarios concerning junction performance and properties are expected to be robust. Calculation improvements could involve using a more accurate method than PBE-D3(BJ) to model structure and reactivity, significantly increased MD simulation times, and use of improved NEGF techniques. In particular, the PBE method used in the NEGF calculations underestimates bandgaps and poorly treats image charges;^80^ the use of empirical modifications or more advanced and expensive methods^77,80,98^ could be used to reduce this effect, if warranted.

A significant difference found between the properties of metal – molecule – metal junctions and the silicon – S(CH_2_)_6_S – silicon ones considered herein is that the conductivity mechanism appropriate to STMBJ experiments differs from that appropriate to blinking experiments. The different experiments realise different junction atomic structures, a feature that produces an often small effect on conductance, whereas the effects on silicon junctions are profound.

The silicon STMBJ junction model most similar to the analogous blinking-experiment model involves fully H-terminated tips, but this is predicted to be insulating rather than conductive. The conductance of STMBJ junctions could only reasonably be modelled when chemical models included silicon junctions with surface dangling bonds. Their conductance is predicted to be very sensitive to the location of the dangling-bonds within the silicon band gap, making them also very sensitive to nuclear structure and associated thermal motion. All dangling-bond chemical scenarios considered herein as models for STMBJs led to features consistent with the primary experimental observations, but the details varied considerably. The minimum level of calculation needed to produce qualitatively reasonable results would appear to be simple bare tips made following the fracture of STM-tip and substrate, as considered in Fig. 5, with sophisticated models like the reformed SAMs considered in Fig. 6 being the next level of improvement.

Using metal junctions like gold, the conductance measured using STMBJ experiments shows greater variability than that measured using blinking experiments, but the results are qualitatively similar. That analogous experiments give very different results using silicon, demonstrating STMBJ conductance ranges that are ten times broader than those obtained from blinking experiments, is explained by the calculations in terms of the necessary presence of remnant silicon surface dangling bonds after cleavage of fused silicon units and subsequent reaction with the solute. It is by manipulating the silicon dangling bonds that some junctions can conduct better than do analogous gold junctions. This dangling-bond effect therefore presents a new way by which molecular-electronic devices could be manipulated and controlled. Added to this is the result apparent from the calculations that circuits can be engineered by varying the silicon doping. These opportunities present many ways by which future devices could be designed.

Overall, the properties of silicon – single molecule – silicon circuits are found to be very different to the traditional metal – single molecule – metal circuits. To expand on this, the potential properties of silicon P–N junctions bridged by molecules are of great interest, and there is an immediate need for their construction and characterisation.

## Methods

### (a) Geometries of model clusters

Initial structures were obtained by taking two H-terminated tip-like clusters Si_10_H_21_, each with one silicon dangling bond site, and bridging S(CH_2_)_6_S between them. The outside rows of Si atoms were frozen parallel at distance *d*′ (see ESI† Fig. S2) ranging from 6.8 Å to 28.0 Å apart. These structures were then optimized by Gaussian-16 (ref. 99) using the B3LYP density function,^100^ the 6-31G* basis set,^101^ and the D3(BJ) dispersion correction.^102^

### (b) Geometries at 0 K of 2D-periodic Si – S(CH_2_)_6_S – Si junctions

2D-periodic models for Si – molecule – Si junctions embody the molecule attached to two slabs representing bulk silicon. A total of 4 layers of Si(111) atoms were used in each slab, with the outside edges H-terminated to mimic the bulk material. The outer two rows on each slab were frozen in all calculations, with the distance *d* (Fig. 3–6) between the second-row silicon atoms in the top and bottom slabs reported as a measure of the inter-electrode vertical separation. A (3 × 3) model of the surfaces was used containing 18 silicon atoms per row, with a lattice parameter of 3 × 3.826862 Å (bulk Si–Si bond length 2.34346 Å). At least 15 Å of vacuum region was placed between each vertical image of the system. Treatment of the inner surfaces varied with sample: sometimes atoms were left bare, sometimes H-terminated, sometimes regular Si tips were added to the innermost layer, and sometimes irregular-shaped tips formed by breaking apart a single piece of silicon were used. Also, sometimes only one slab was used and chemical reaction and activation energies calculated for the attack of various solvent or solute molecules on silicon dangling bonds.

2D-geometry optimisations were performed by VASP^103,104^ 5.4.1 using the PBE density functional^105^ with the D3(BJ) dispersion correction^102^ applied in the in-layer directions. Control parameters used include: normal precision, an energy convergence criterion of 10^−5^ eV, and a gradient convergence of 0.01 eV Å^−1^. Thermal broadening of the occupied orbital energies was performed at an electronic temperature corresponding to 0.2 eV. Dipole corrections were not employed.

### (c) Molecular dynamics studies of 2D-periodic Si – S(CH_2_)_6_S – Si junctions

Molecular dynamics simulations at 300 K were performed on various junctions by VASP^103,104^ 5.4.1 using the PBE density functional^105^ with the D3(BJ) dispersion correction^102^ applied in the in-layer directions. Sample and unit cell details were as per the 2D calculations performed at 0 K (see above). Control parameters used include: low precision, the “very fast” algorithm, an energy convergence criterion of 10^−4^ eV, and a time step of 1 fs (results using 2 fs were insignificantly different, but 3 fs proved unacceptable), and a trajectory length of 1 ps. All of the data was used in calculating averaged properties, except that configurations were sampled after every 16 fs for conductivity analysis.

### (d) NEGF calculations

Electronic-transport properties were calculated with the NEGF technique,^74^ as implemented in Nanodcal package (version 2016).^74,106^ The molecular electronic junction was divided into three regions, bottom electrode, contact region, and top electrode. The contact region includes parts of the physical electrodes (4 layers of silicon or 3 layers of gold), possible additional electrode atoms above the surface, and the bridging molecule. All NEGF calculations of molecular junction electron transport properties were performed self-consistently at *T* = 300 K electronic temperature. The contact region Green's functions are used to compute electronic transmission spectra and, consequently, molecular junction conductivities. The PBE density functional was used, along with double-zeta with polarization basis set, *k*-space grids of 3 × 3 × 1, and 50 a.u. energy cutoff for the real-space grid. Pseudopotentials for silicon atoms were fitted separately to reproduce the required experimental phosphorous (N-type) and boron (P-type) doping, utilizing DFT bulk silicon calculations. These are set to mimic doping levels of 1.15 × 10^20^ atoms cm^−3^ for P-doped silicon, and 7 × 10^19^ atoms cm^−3^ for N-doped.

## Data availability

The most significant data concerns the atomic coordinates and calculation properties pertaining to the 80 structural images shown in figures in the main text and ESI, for which full details are provides in ESI. Additional information is available from the authors.

## Author contributions

J. R. R. and J. Y. performed the DFT calculations, whilst D. S. K. performed the NEGF calculations. J. R. R., N. D., and D. S. K. designed the research.

## Conflicts of interest

There are no conflicts to declare.

## Supplementary Material

SC-012-D1SC04943G-s001

SC-012-D1SC04943G-s002
