# Supplementary material for: Silicon – single molecule – silicon circuits
Source: Chem Sci. 2021 Oct 29;12(48):15870–81. doi: 10.1039/d1sc04943g (PMC8672724; doi:10.1039/d1sc04943g)
Supplement: SC-012-D1SC04943G-s002 [file SC-012-D1SC04943G-s002.pdf]

Electronic Supplementary Material (ESI) for Chemical Science.
This journal is © The Royal Society of Chemistry 2021

Silicon – single molecule – silicon circuits

Jeffrey R. Reimers[1,2], Junhao Yang[1], Nadim Darwish[3], and Daniel S. Kosov[4]

1. International Centre for Quantum and Molecular Structures and School of Physics, Shanghai University, Shanghai 200444, China
2. 3. School of Mathematical and Physical Sciences, University of Technology Sydney, NSW 2007 Australia, Email Jeffrey.Reimers@uts.edu.au

2. School of Molecular and Life Sciences, Curtin Institute of Functional molecules and Interfaces, Curtin University, Bentley, WA 6102, Australia. Email nadim.darwish@curtin.edu.au.

4. College of Science and Engineering, James Cook University, Townsville, QLD 4811, Australia, Email daniel.kosov@jcu.edu.au

Electronic Supporting Information

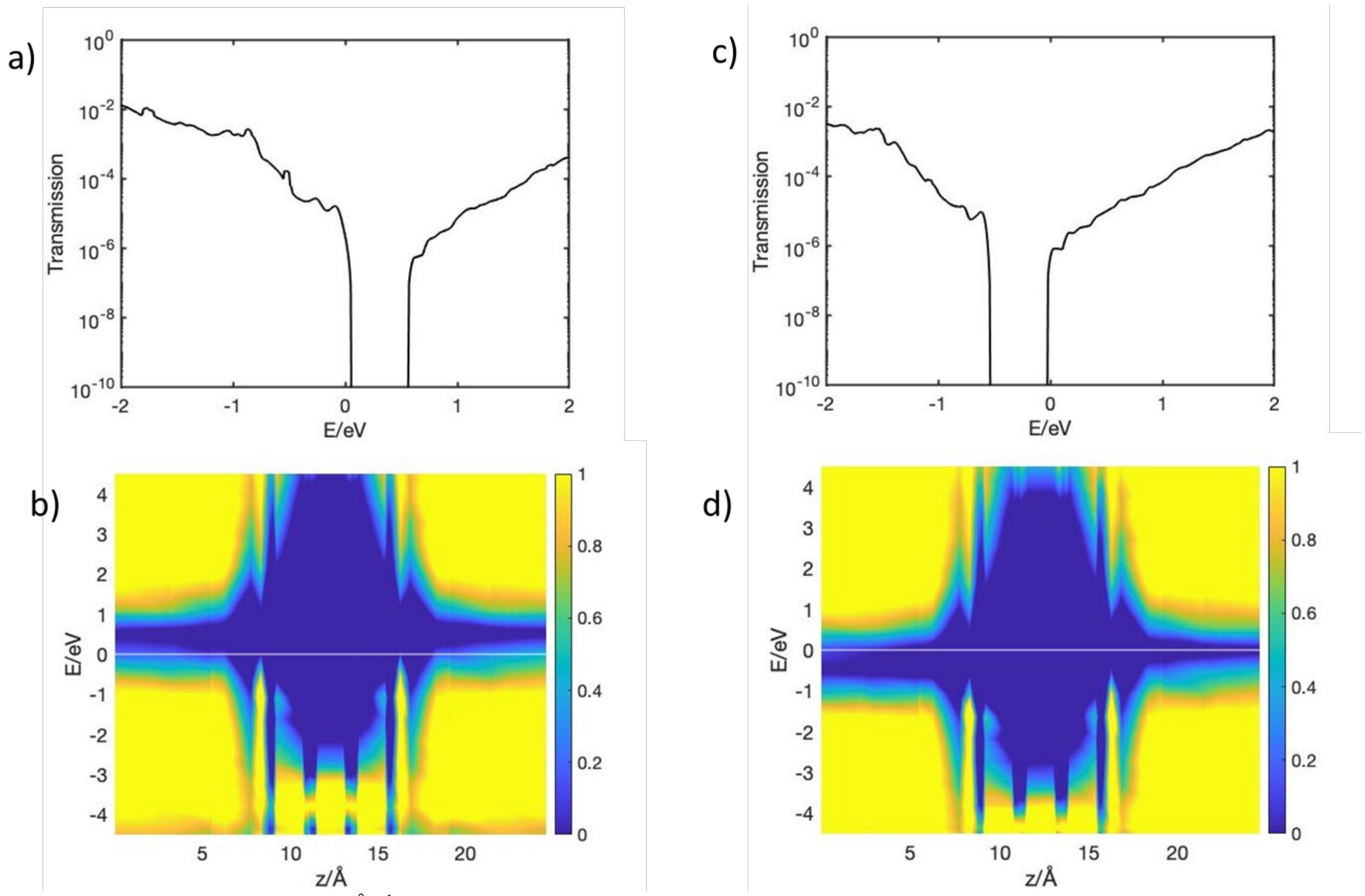

Fig. S1. Calculated PDOS (in (eV Å)$^{-1}$) from NEGF calculations of single-molecule conductivity for two silicon electrodes bridged by $S(CH_2)_6$ in the configuration modelling blinking experiments shown in Fig. 2. This involves a SAM at 1:9 coverage bridging Si(111)-H surfaces. (a) and (b) are transmissions and projected DOS computed for P-doped silicon, whilst (c) and (d) shows the results for N-doped silicon. As the molecular HOMO level lies much closer to the Fermi energy in P-doped silicon than the LUMO is to the Fermi energy for N-doped silicon, the calculated conductivity for P-doping is much higher (see main text).

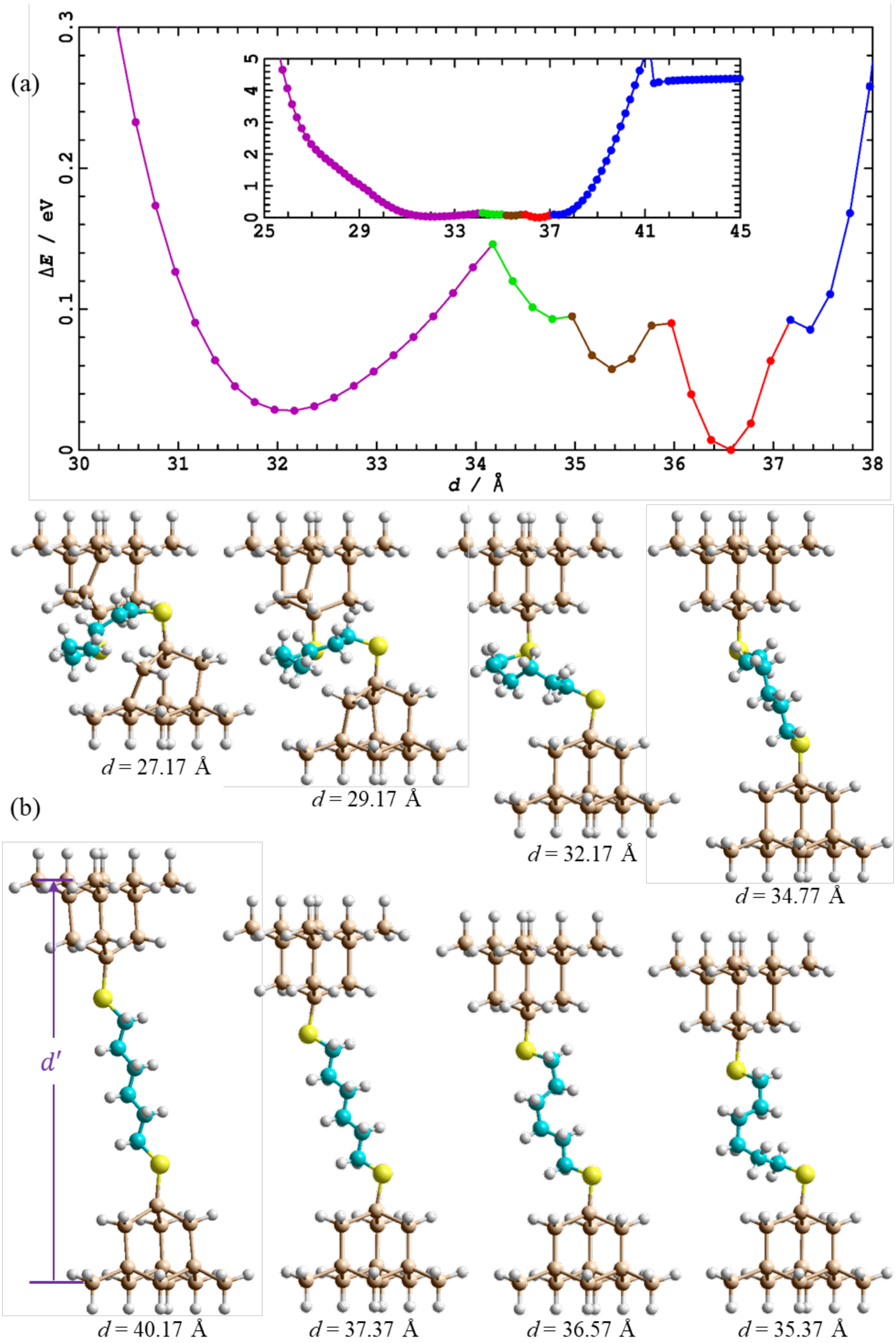

Fig. S2. (a) Potential-energy surface for a cluster model in which two $Si_{10}H_{21}$ clusters represent the silicon surfaces, bridged by $S(CH_2)_6S$. The clusters are constrained with their outside planes parallel, with frozen shape. The vertical distance d' between the outer layers is therefore constrained during the optimisations. When the resultant optimised geometries are imbedded into 2D periodic models frozen from their 3rd layer out (see Fig. 3), the constrained vertical distances increase to $d = d' + 17.171$ Å, and to be consistent with the distance scales presented elsewhere, energy is shown as a function of d. The coloured regions depict different alkane-chain conformations. (b) Optimised structures at selected geometries, later used for optimisation in 2D models.

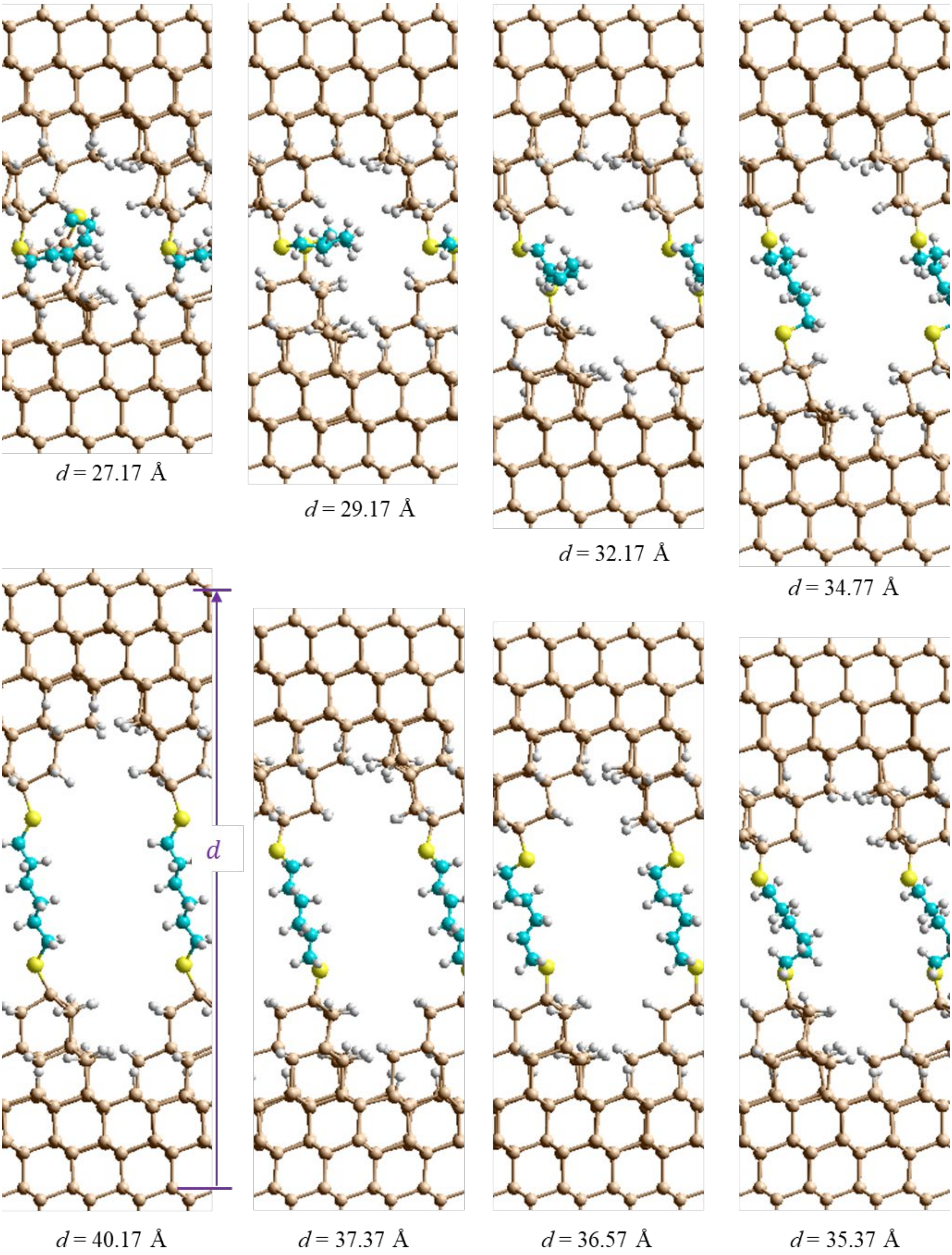

Fig. S3a. Modelling the STMBJ experiment assuming regular H-terminated silicon tips form on each electrode, showing 2D (3×3) models of two flat Si(111)-H surfaces with two added H-terminated Si layers in the shape of a tip, spanned by $S(CH_2)_6S$, at 2nd-outer-layer to 2nd-outer-layer distance *d*. Shown are structures after 1 ps of MD following insertion of the structures from Fig. S2 inside Si(111)-H slabs and initial optimisation. Si- brown, S- yellow, C- cyan, H- white.

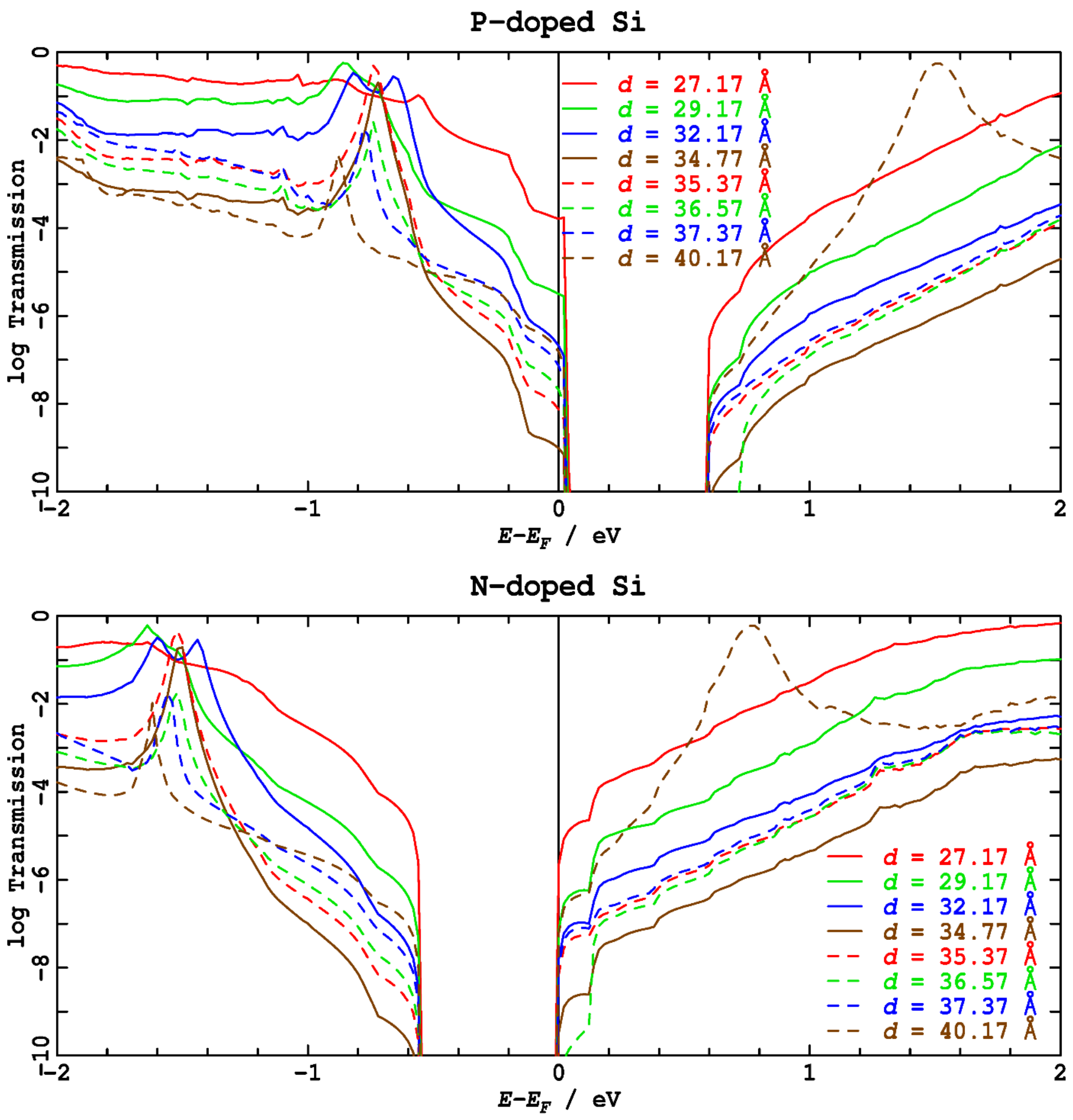

Fig. S3b. Transmission as a function of energy away from the Fermi energy, from NEGF calculations, for the $T = 300$ K sample structures from Fig. S3a, pertaining to STMBJ conditions assuming regular H-terminated silicon tips form on each electrode for two flat Si(111)-H surfaces with two added H-terminated Si layers in the shape of a tip, spanned by $S(CH_2)_6S$. The conductance at zero voltage is determined from the transmission at $E - E_F = 0$, a region in which the transmission drops extremely rapidly. As a result, details of the calculated conductance become very sensitive to model and to the density of states at the fermi energy. For P-type silicon, the sensitivity is much less than for N-type silicon. The rapid fall in transmission as the junction gap parameter is extended from $d = 27.17$ Å to 34.77 Å is caused by the exponential decrease in silicon-silicon through-space tunnelling with distance; beyond this distance, the straightening of the alkyl chain increases the transmission until the extensive bond extension at 40.17 Å acts to reduce it.

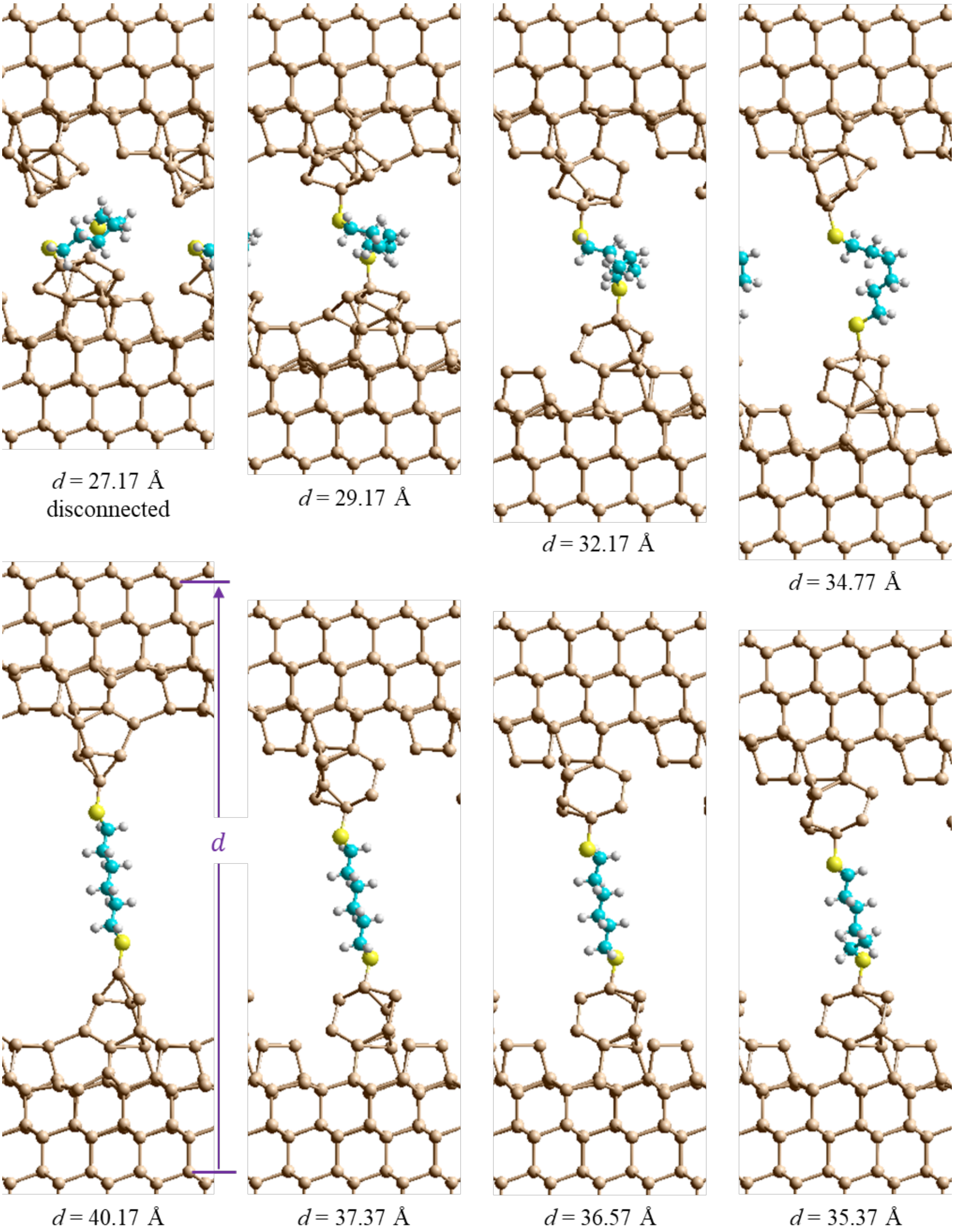

Fig. S4. Modelling the STMBJ experiment assuming unterminated silicon surface layer and extending tips form on each electrode (starting configurations are those from Fig. S3a deprotonated), showing 2D (3×3) models optimised at 0 K, with the tips spanned by $S(CH_2)_6S$, at 2nd-outer-layer to 2nd-outer-layer distance *d*. Si- brown, S- yellow, C- cyan, H- white. At $d = 34.7$ Å, the molecule desorbed from the upper tip to bind twice to the lower tip, destroying any through-molecule conductivity.

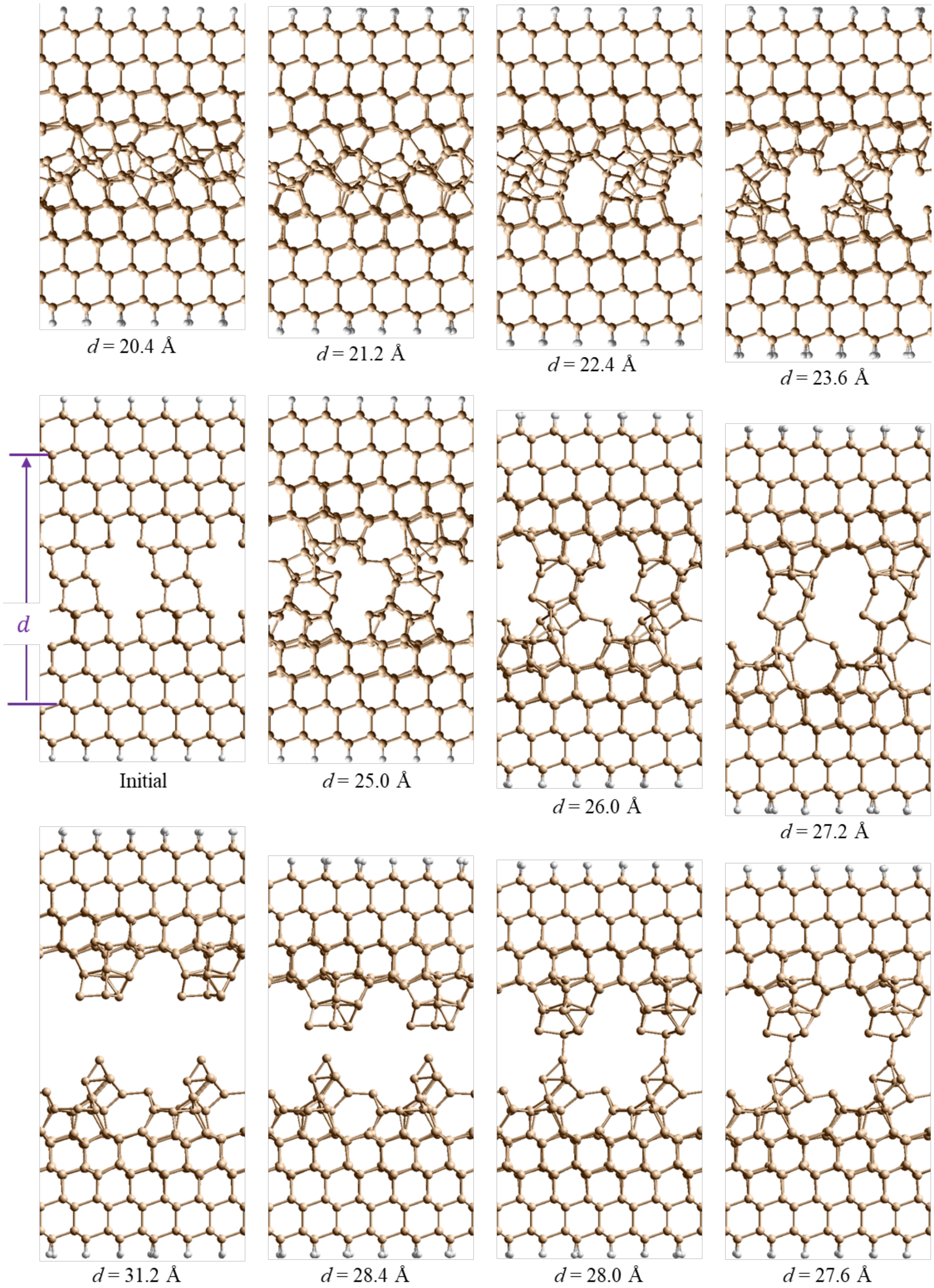

Fig. S5a. Simulation of the breaking of an amorphous junction formed after two pieces of silicon collide, following MD simulations for 1 ps at 300 K. The initial structure is a regular 11-row Si(111) lattice from which atoms have been removed from the centre at d = 25 Å. This structure is first heated to 300 K and then both pulled apart and compressed in, using steps of 0.2 Å, running MD after each increment. The structure at d = 20.4 Å has the density of bulk silicon. Si- brown.

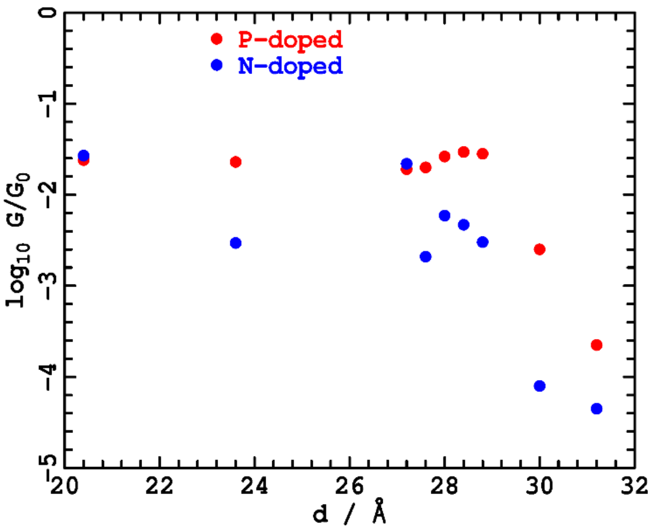

Fig. S5b. Average conductance during the fracture of the silicon break junction (Fig. S5a) from 1 ps MD simulations at 300 K. The calculated conductance of a crystalline slab of P-doped Si, using 3 layers in the "junction" region is 0.24 G_0. This corresponds to a resistivity of 0.0066 Ωcm, near the observed value at that level of doping of 0.001 Ωcm. The reduced value here at the crystal density (d = 20.4 Å) owes to the finite size and amorphous nature of the STMBJ junction region; conductance decreases exponentially following junction rapture (near d = 28.4 Å). Owing to large conductance fluctuations on the fs timescale, the extent of MD averaging is mostly insufficient for the evaluation of quantitatively accurate results.

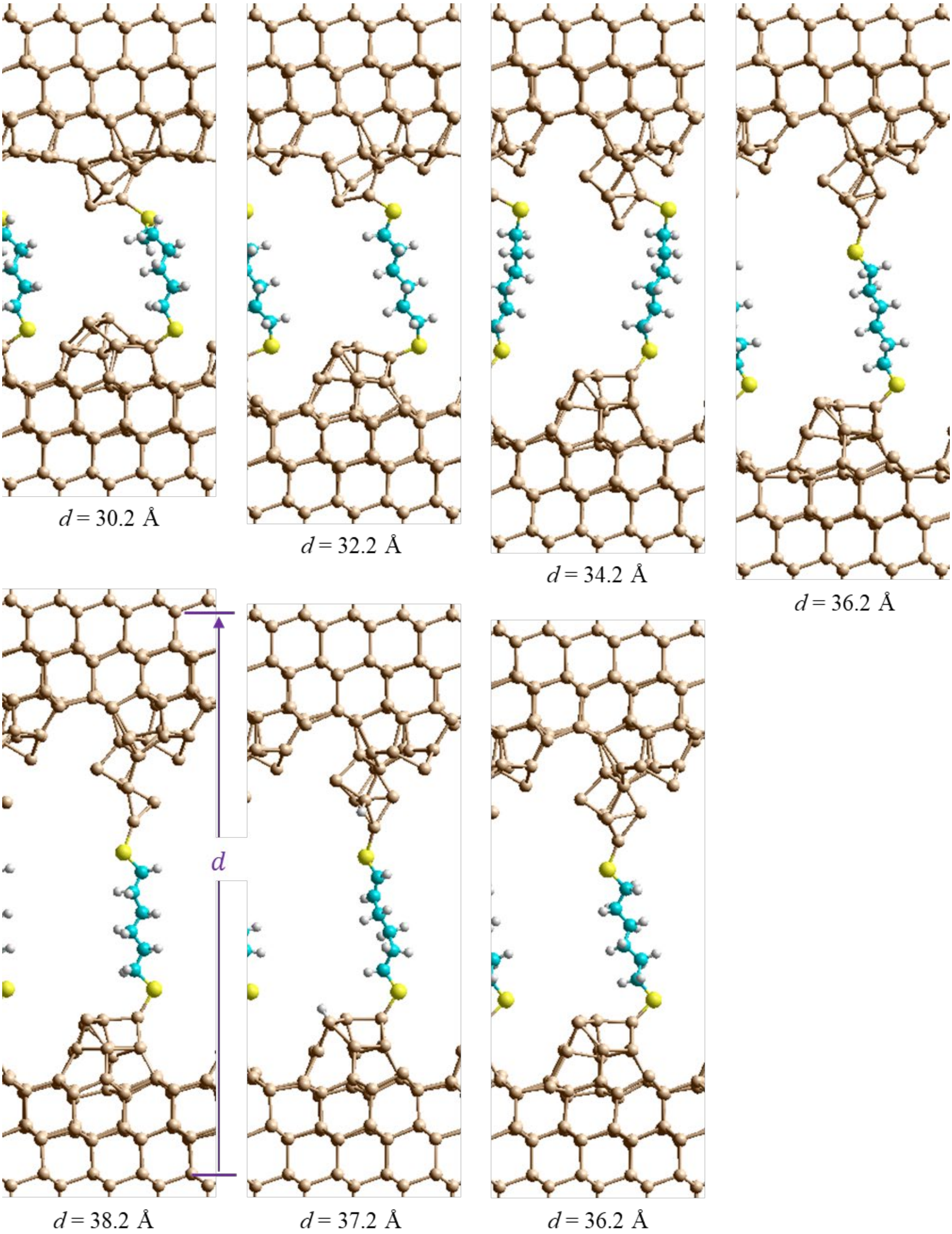

Fig. S6. Selected optimised structures at T = 300 K for $S(CH_2)_6S$ bound between the tips formed after fracturing a silicon slab (see Fig. S5). Si- brown, S- yellow, C- cyan, H- white. A reorientation occurred at d = 36.2 Å, and hence the data is presented as two independent series (top row and bottom row).

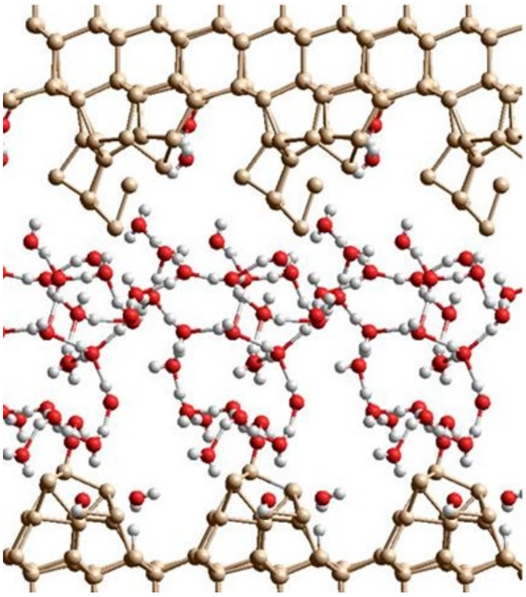

Fig. S7. Simulation of liquid water between the broken Si(111) tips (see Fig. S5), at ca. 80% of actual liquid density to accelerate processes, after 1 ps of MD, showing chemical reactions with the surface. Si- brown, O- red, H- white.

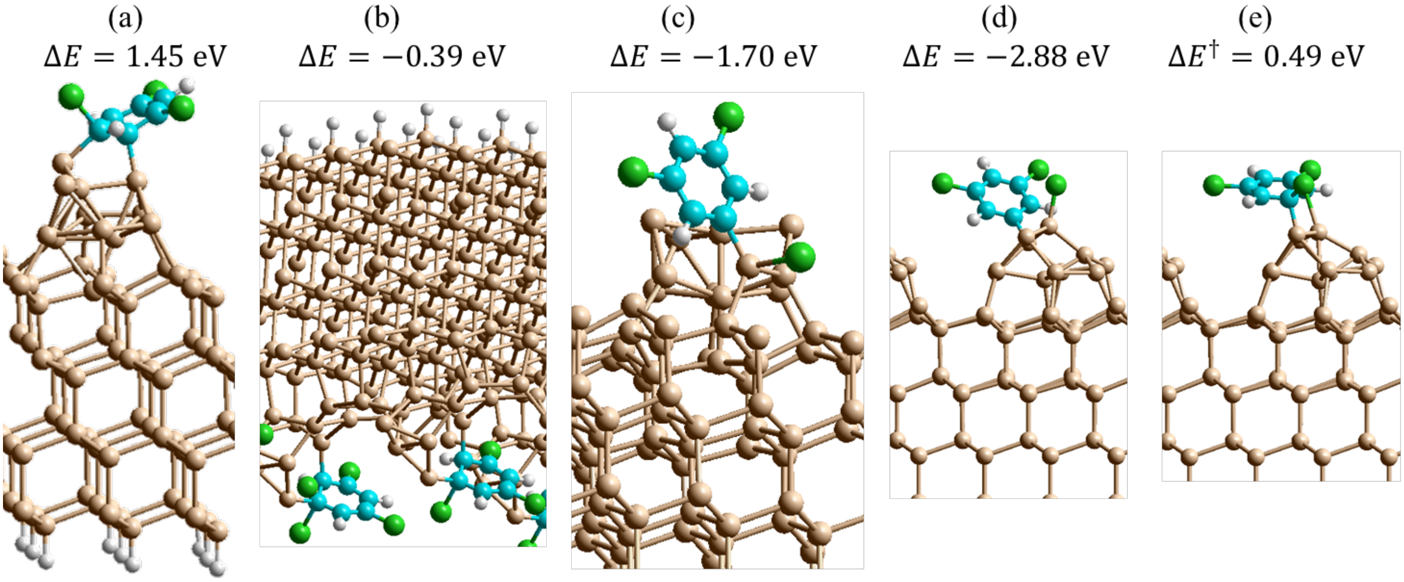

Fig. S8. Reactions of 1,3,5-trichlorobenzene (TCB) solvent with the upper and lower tips formed in the STMBJ simulation (see Fig. S5). (a)-(b) Addition of two Si radicals across a CC bond; (c)-(d) Insertion of two Si radicals to break a C-Cl bond; (e) Lowest-energy transition state found for any reaction, this being for the insertion of the two Si radicals to break a C-Cl bond. Si- brown, S- yellow, C- cyan, H- white, Cl- green.

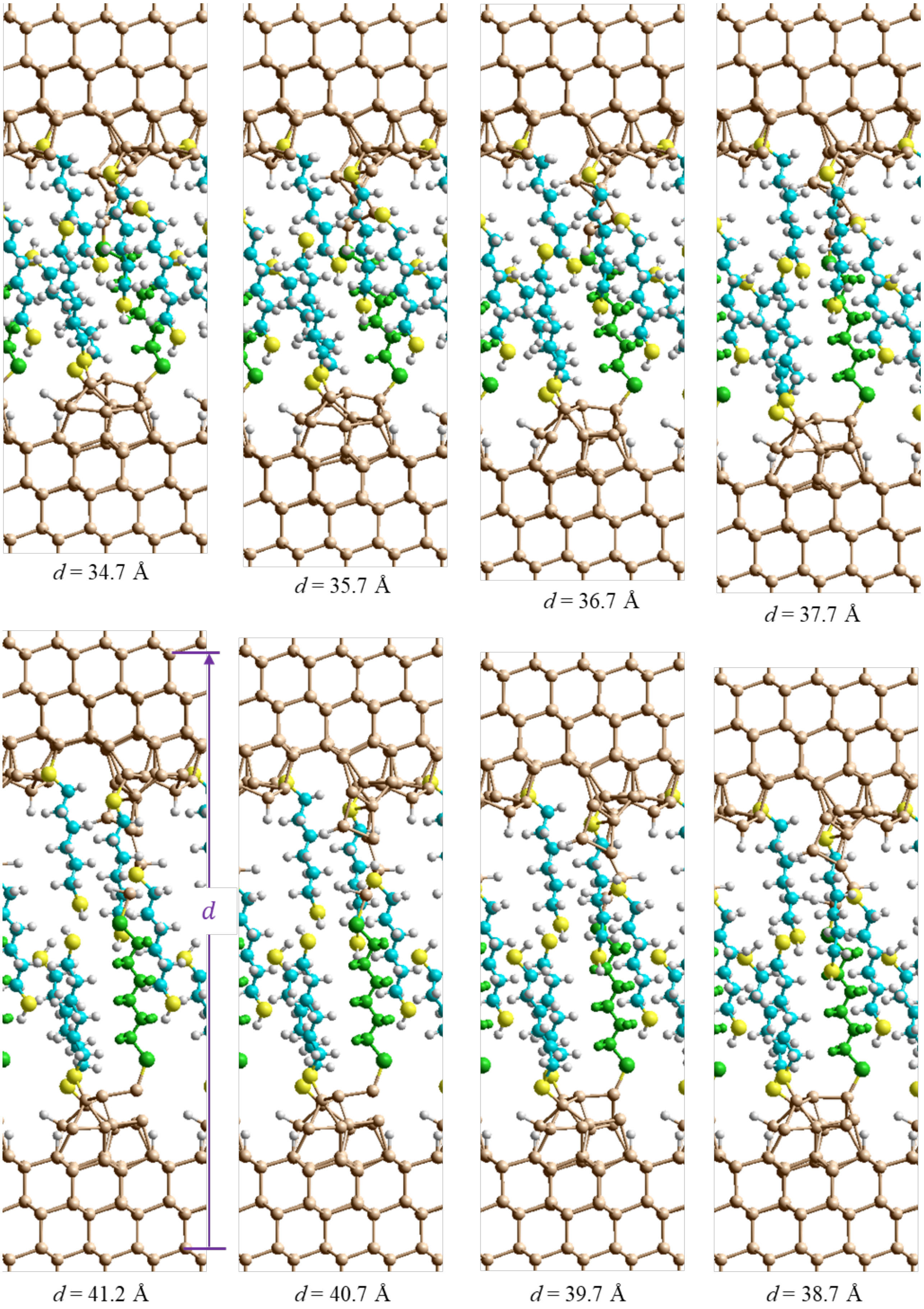

Fig. S9a. Selected optimised structures at $T = 0$ for the "l" series with six solvent molecules reacted with the bare silicon tips (see Fig. S5) to bind both S and H to the surface. Some silicon radical sites remain unpassivated. The SAM coverage per cell is 6:9, less than the 7:9 value observed for flat SMAMS at high density. At $d = 41.2$ Å, a top-electrode Si-Si bond breaks leaving one bonded Si-bridged dimer as $-S(CH_2)_6S-Si-S(CH_2)_6SH$. Si- brown, S- yellow, C- cyan, H- white, bridging molecule- green.

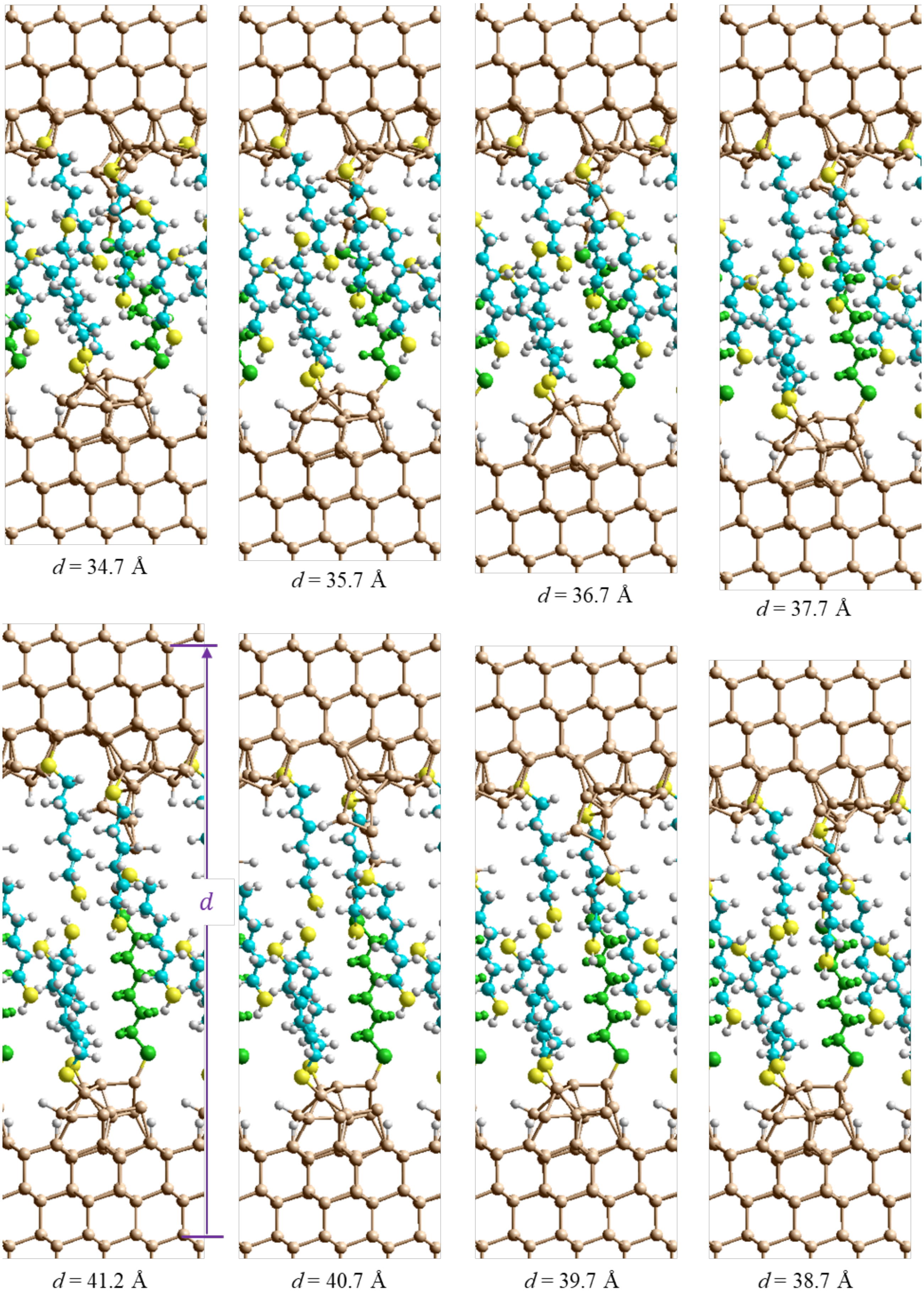

Fig. S9b. Selected optimised structures at $T = 0$ for the "m" series with six solvent molecules reacted with the bare silicon tips (see Fig. S5) to bind both S and H to the surface. Some silicon radical sites remain unpassivated. The SAM coverage per cell is 6:9, less than the 7:9 value observed for flat SAMS at high density. At d = 41.2 Å, a top-electrode Si-Si bond breaks leaving one bonded molecule as –S(CH_2)S–SiH. Si- brown, S- yellow, C- cyan, H- white, bridging molecule- green.

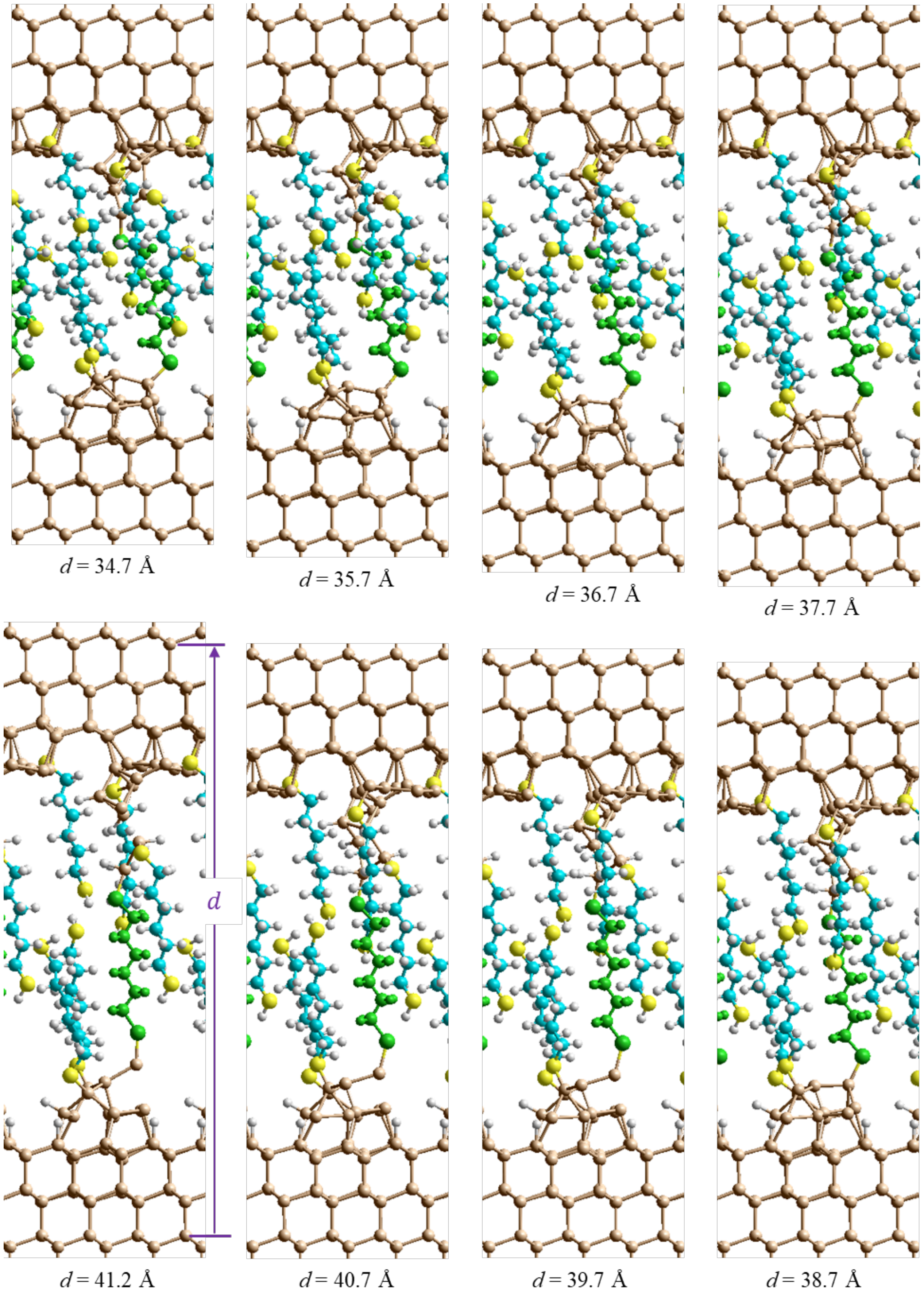

Fig. S9c. Selected optimised structures at $T = 0$ for the "n" series with six solvent molecules reacted with the bare silicon tips (see Fig. S5) to bind both S and H to the surface. Some silicon radical sites remain unpassivated. The SAM coverage per cell is 6:9, less than the 7:9 value observed for flat SAMS at high density. At $d = 41.2$ Å, a top-electrode Si-Si bond breaks leaving one bonded Si-bridged dimer as $-S(CH_2)_6S-SiH=SiH-S(CH_2)_6SH$. Si- brown, S- yellow, C- cyan, H- white, bridging molecule- green.

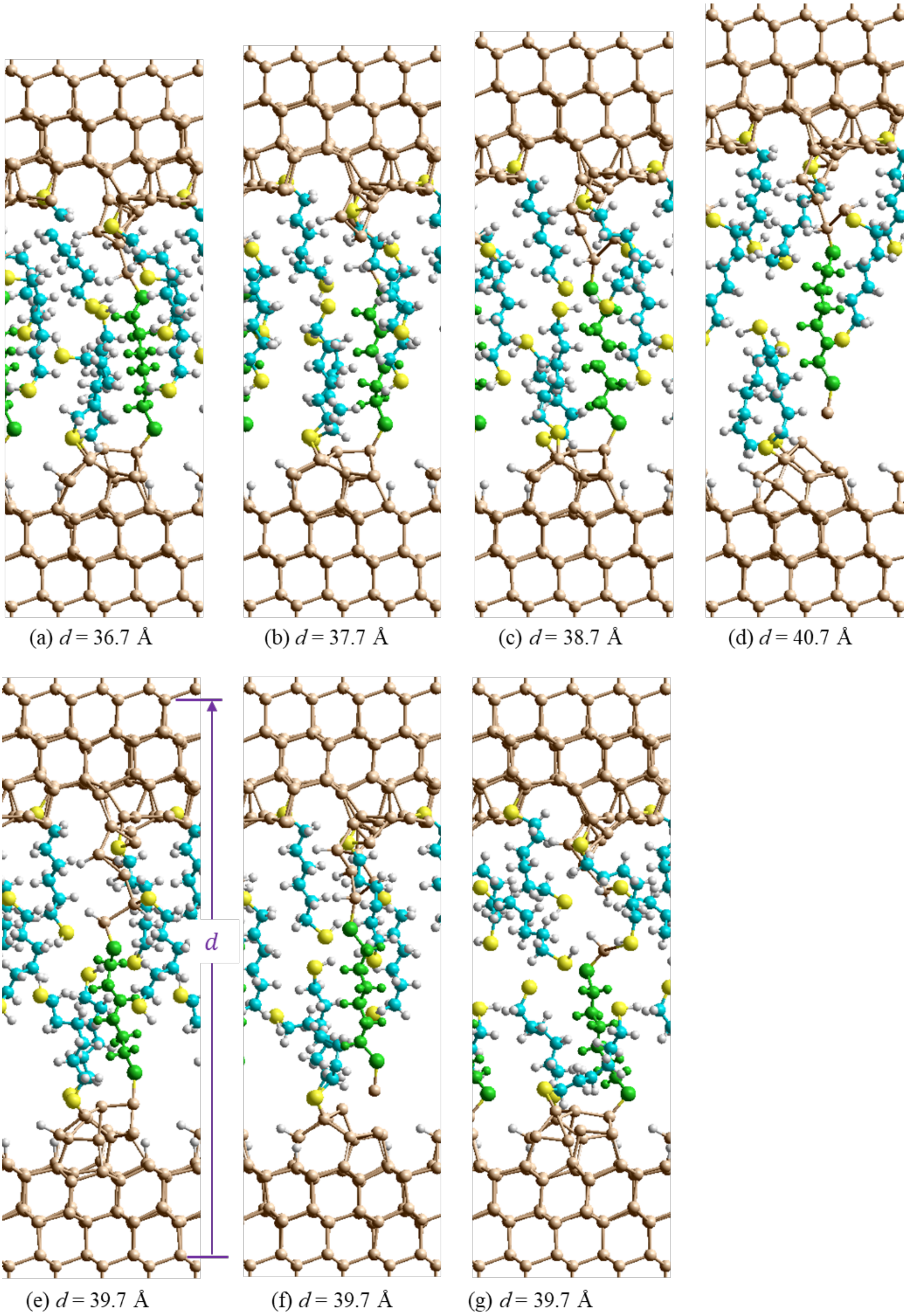

Fig. S10. Snapshots from the MS simulations at 300 K for the "n" series SAMs formed by reacting $HS(CH_2)_6SH$ in solution with the fractured silicon tips from Fig. S2. (a)-(c) Order dynamics prevails on the 1 ps timescale for $36.7\ \text{Å} \leq d \leq 38.8\ \text{Å}$. (d) At $d = 40.7$ Å, dynamics it mostly ordered, by large reversible displacements, like the one shown in which a critical Si-Si bind breaks. (e)-(g) Snapshots for $d = 39.7$ Å, revealing for (e): large structural changes from the most common structural type; for (g): reversible breaking a lower Si-Si bond; and (h): irreversible reaction in which an broken upper Si-Si bond leads to reaction with a tail-group SH from another ligand in the SAM to form a SH-SiH-SH bridge as possibly an artefact owing to the low SAM density that results at large extension, with reactions of remaining silicon dangling-bond sites with solvent or solute being likely to occur to increase the density. Si- brown, S- yellow, C- cyan, H- white, bridging molecule- green.

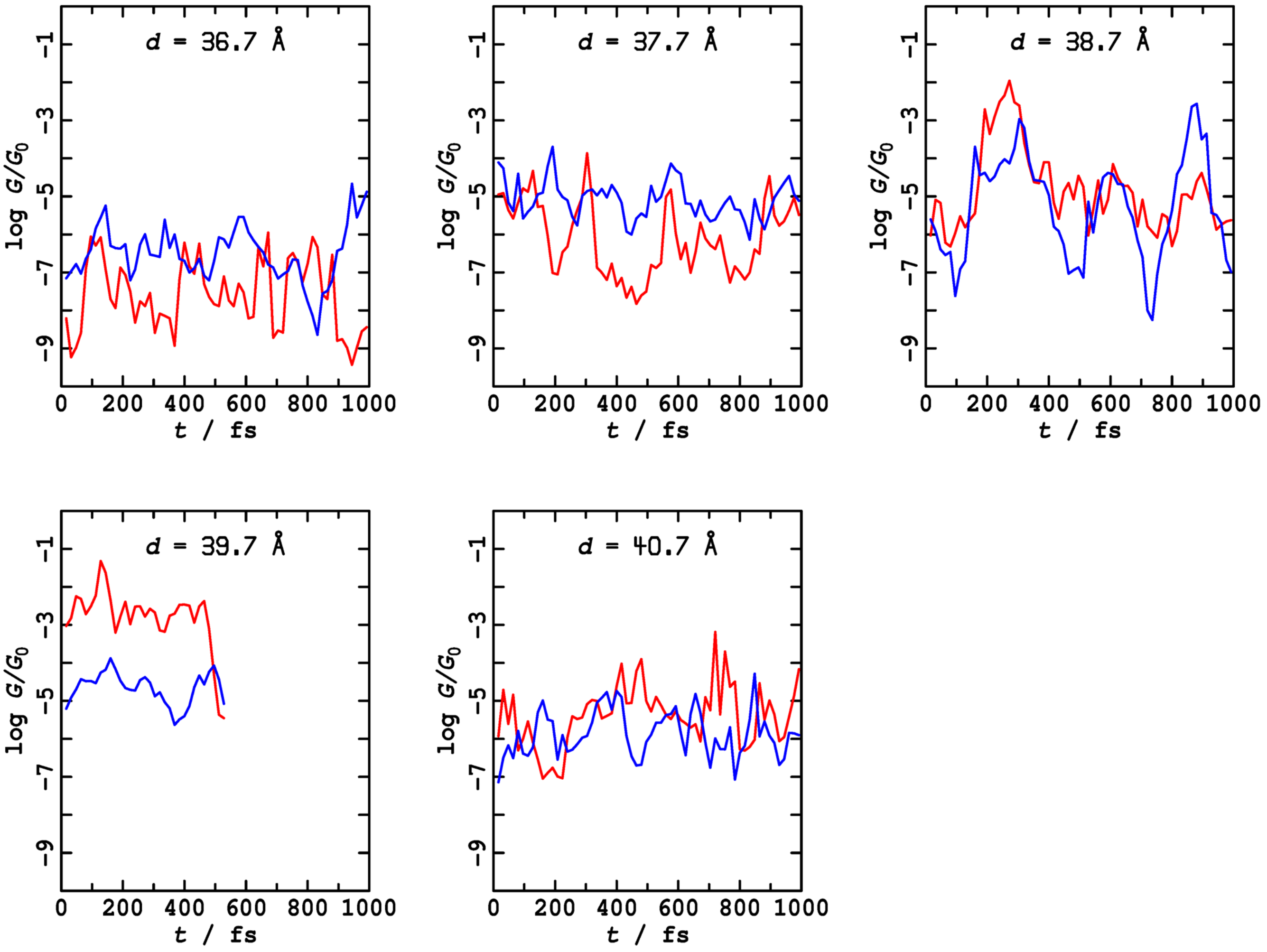

Fig. S11. Variation of the conductivity as a function of time throughout the MD simulation of the "n" series SAMs formed by reacting $HS(CH_2)_6SH$ in solution with the fractured silicon tips from Fig. S2. Red: P-type silicon, blue: N-type silicon.

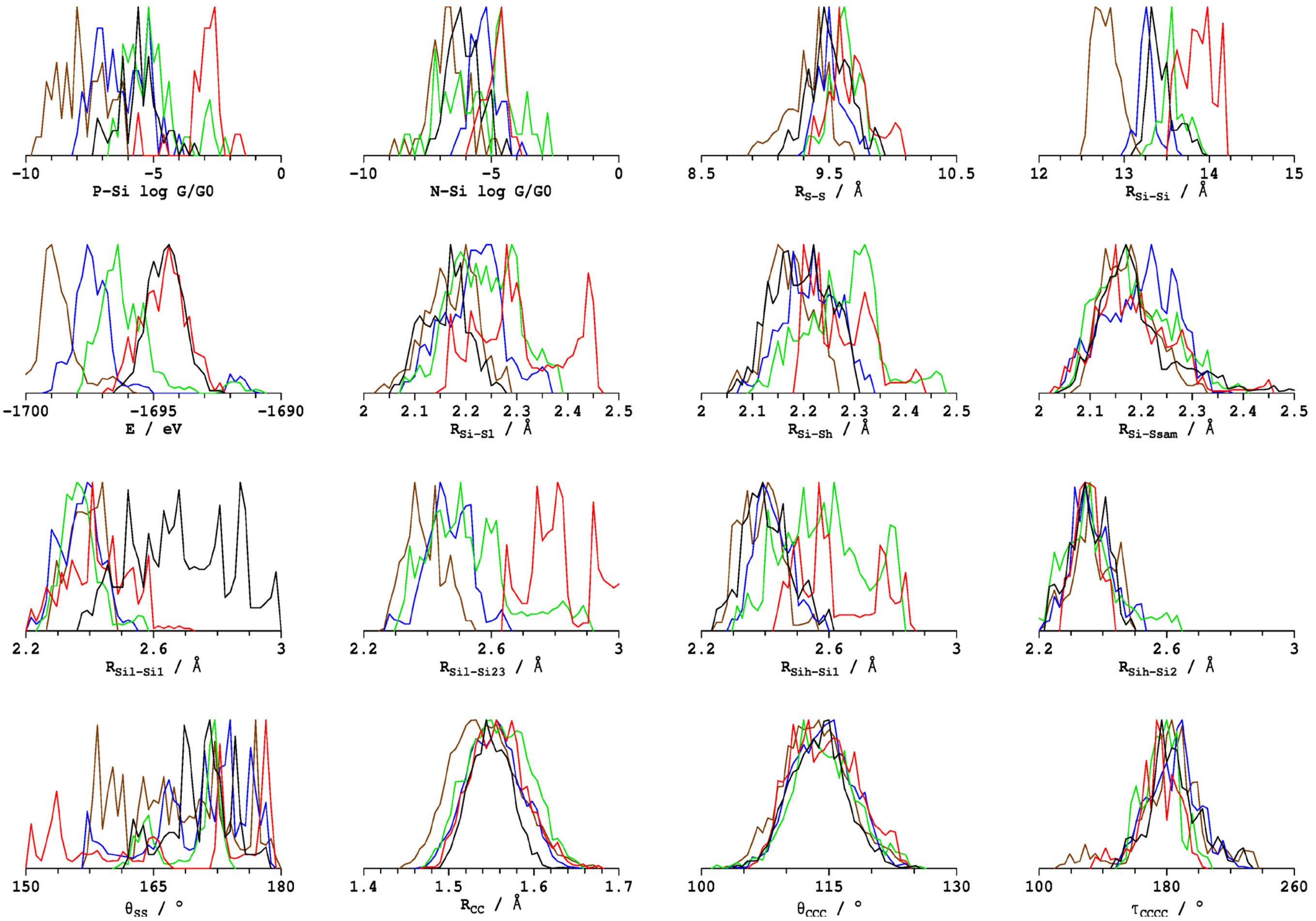

Fig. S12. Probability of occurrence of different properties evaluated from the MD simulations at 300 K for the "n" series SAMs formed by reacting $HS(CH_2)_6SH$ in solution with the fractured silicon tips from Fig. S2. This includes: the total energy profile E, The NEGF conductances G for P-type Si and for N-type Si, the bridging molecule S-S distance $R_{\mathrm{S-S}}$, the related distance between the Si atoms bonded to the S atoms $R_{\mathrm{Si-Si}}$, from amongst these atoms, the lower $R_{\mathrm{Si-Sl}}$ and upper $R_{\mathrm{Si-Sh}}$, bond lengths, the analogous average bond length for Si-S on a molecule in the MAS that does not bridge the lower and upper electrodes $R_{\mathrm{Si-Ssam}}$, the three Si-Si bond lengths tethering the lower Si onto the electrode $R_{\mathrm{Sil-Si1}}$, and the average of $R_{\mathrm{Sil-Si2}}$ and $R_{\mathrm{Sil-Si3}}$, two corresponding Si-Si bond lengths for the upper electrode $R_{\mathrm{Sih-Si1}}$ and $R_{\mathrm{Sih-Si2}}$, the angle of the S-S vector of the bridging molecule to the z-direction, θ_{SS}, and the average CC bond lengths R_{CC}, CCC bond angles θ_{CCC}, and CCCC torsional angles τ_{CCCC}. Brown- $d = 36.7$Å, blue- $d = 37.7$ Å, green- $d = 38.7$ Å, red- $d = 39.7$ Å, black- $d = 40.7$ Å.
